# Improving the stability and color purity of a BT.2020 blue multiresonance emitter by alleviating hydrogen repulsion

**DOI:** 10.1126/sciadv.adh1434

**Published:** 2023-05-12

**Authors:** Xiang Wang, Lu Wang, Guoyun Meng, Xuan Zeng, Dongdong Zhang, Lian Duan

**Affiliations:** ^1^ Key Laboratory of Organic Optoelectronics, Department of Chemistry, Tsinghua University, Beijing 100084, China.; ^2^ Center for Flexible Electronics Technology, Tsinghua University, Beijing 100084, China.

## Abstract

Stable deep blue multiresonance emitters with small full width at half maximum (FWHM) are attractive for wide color-gamut organic light-emitting diodes (OLEDs). However, the steric repulsion from the spatially close hydrogens would twist the multiresonance skeletons, causing spectral broadening and molecular instability issues. Here, we strategically introduce a mesitylboron locking unit into a carbazole-embedded multiresonance model emitter, alleviating the hydrogen repulsions and also strengthening the para-positioned weak carbon-nitrogen bond in anionic states. An emission peaking at 452 nm with an FWHM of merely 14 nm and nearly BT.2020 blue chromaticity coordinates are obtained in toluene, affording a high maximum external quantum efficiency of 33.9% in a sensitizing device. Moreover, an impressive LT97 (time to decay to 97% of the initial luminance) of 178 hours at a constant current density of 12 mA/cm^2^ was achieved in a stable device with a small *y* coordinate of 0.057, nearly 20 times longer than the model emitter with even a substantially red-shifted emission.

## Teaser

A stable BT.2020 blue multiresonance emitter was achieved by adopting a mesitylboron locking strategy.

## INTRODUCTION

In the field of organic light-emitting diodes (OLEDs), conceptual advancements in the organic emitting materials would usually revolutionize this technology and lastly pave the way toward commercialization. The replacement of fluorescent dopants by phosphorescent and thermally activated delayed fluorescence (TADF) emitters quadruples internal/external quantum efficiency via harnessing the triplet excitons in different mechanisms (*1*–*3*). Despite the successes in green and red regions, efficient and stable blue phosphorescent and TADF OLEDs remain a formidable challenge as the high emission energy could lead to material degradation (*4*). Moreover, the charge transfer (CT) nature of donor-acceptor–type TADF emitters and the hybridization of metal-to-ligand CT and ligand-centered emission of phosphorescent emitters inevitably promote structural relaxations at excited states and induce broadened emission spectra with full width at half maximum (FWHM) of more than 50 nm, which require the adoption of additional color filter and/or optical microcavity in practical applications and result in efficiency loss (*5*–*8*). Therefore, developing intrinsic narrowband blue emitters with high efficiency and stability is vital in the OLED community.

The recently emerged heteroatoms embedded polycyclic aromatic hydrocarbons (PAHs) with multiple resonance (MR) characteristics provide an opportunity to achieve the above objective (*9*–*12*). To be more specific, the MR effect induces an alternating pattern of the frontier molecular orbitals on single atoms of PAHs, greatly suppressing structural relaxation and vibronic coupling between the ground and excited states to afford intrinsic small FWHMs (*13*–*19*). The use of such narrowband emitters could avoid the use of additional color filters and also have the potential to achieve the latest Broadcast Service Television 2020 (BT.2020) standard for blue color with relatively smaller onset and peak energies than the conventional fluorescent emitters, thus avoiding the ultraviolet region part and benefiting both device stabilities and eye health (*20*, *21*). The most judicious molecular design strategy for MR emitters is to embed mutually ortho-positioned electron-deficient boron and electron-rich nitrogen atoms into a triangulene core structure, first pioneered by Hatakeyama *et al.* (*22*) in the well-known DABNA series. Following works have been devoted into modulating molecular structures to optimize the optoelectronic properties and MR emitters having FWHMs in the range of 20 to 40 nm are in the dominate positions now with quite few less than 20 nm. Among them, the most standout molecule should be ν-DABNA, which achieved a narrow FWHM of 18 nm in the electroluminescence (EL) device but with an unsatisfactory Commission Internationale de l’Éclairage *y* coordinate (CIE*_y_*) of 0.11 (*17*). By rationally designing triarylamine-based precursors for π-extension, Yang and coworkers reported a deep blue emitter BN3 displaying an impressive maximum external quantum efficiency (EQE_max_) of 36.3% with an FWHM of 21 nm and CIE*_y_* of 0.07 (*23*). By replacing the two intracyclic nitrogen atoms with oxygen atoms, Yasuda and coworkers developed a deep blue MR emitter BOBO-Z realizing an EQE_max_ of 13.6%, a FWHM of 18 nm and a small CIE*_y_* of 0.04 (*24*). To the best of our knowledge, nearly all blue MR emitters with EL spectral FWHMs of less than 20 nm are ν-DABNA analogs, and the pursuit to further narrow the emission spectra has been slowed down because of limited diversity in molecular skeletons (*25*, *26*). Besides the spectral problems, a more concerned and fundamental but less studied issue is the intrinsic stability of MR emitters, particularly for the deep blue ones. Albeit the fact that the rigid polycyclic aromatic backbone should endow MR emitters with sufficient thermal and chemical stabilities, only few MR molecules have shown decent operation lifetimes in the commercialized triplet-triplet annihilation (TTA)–based device structures with LT97s (time to decay to 97% of the initial luminance) in the range of tens to hundreds of hours at an initial luminance of 200 cd/m^2^. Nevertheless, those lifetimes are still unsatisfied for practical applications, not to mention their relatively large FWHM > 20 nm. The lack of an in-depth understanding on the structure-stability relationship has greatly limited the further improvement of device lifetimes.

**Fig. 1.**
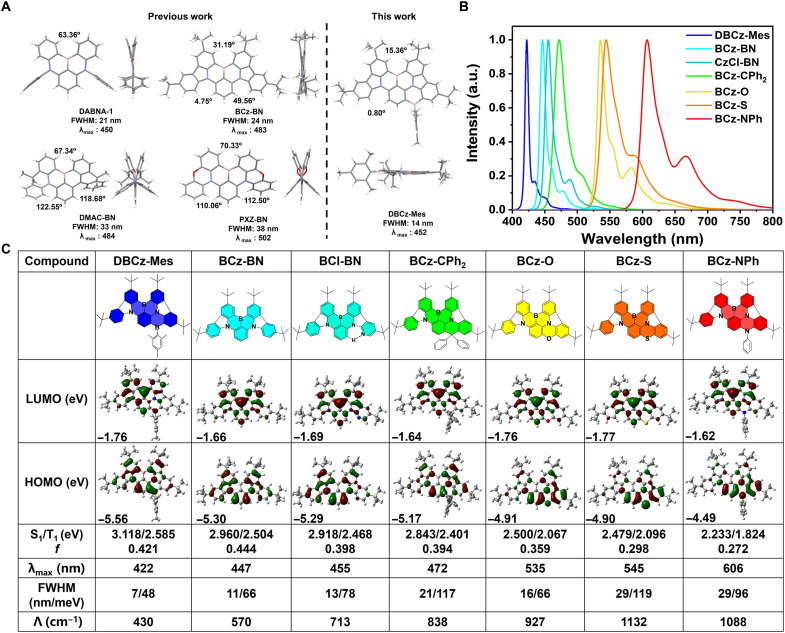
Crystal structures and theoretical calculations. (**A**) Crystal structures of DABNA-1, DMAC-BN, PXZ-BN, BCz-BN, and DBCz-Mes. The numbers represent the torsion angles between the adjacent C─H’s. (**B**) Computer simulated emission spectra of BCz-BN and its locked analogs using the MOMAP software. a.u., arbitrary units. (**C**) Molecular structures, frontier molecular orbital (FMO) distributions, calculated excited states, and emission properties at the PBE0/6-31G(d,p) level for the compounds with different locking methods; λ, reorganization energy.

In theory, the photophysical properties and stability issues of MR emitters should strongly relay on the planarity of their structures. A more rigid and planarized skeleton would benefit to suppress structural relaxation at excited states and thus further narrow the emission spectra (*27*–*28*). Besides, as having been pointed out by Yamaguchi and coworkers, a molecular structure being constrained in a fully planar geometry can effectively improve the stability of triarylboranes by facilitating the delocalization of π electrons to stabilize the reactive empty p orbital of the boron atom (*29*–*32*). One can therefore envision that planarizing the MR core should be effective in simultaneously narrowing FWHMs and enhancing molecular stability. Given that most MR molecules are derived from basic parent structures, we studied the crystal structures of several classical parent MR skeletons containing phenylamine (DABNA-1, FWHM of 21 nm), carbazole (BCz-BN, FWHM of 24 nm), 9,9-dimethyl-acridine (DMAC-BN, FWHM of 33 nm), and phenoxazine (PXZ-BN, FWHM of 38 nm), respectively (*22*, *33*, *34*). Clear steric repulsions from the spatially close hydrogens were observed for all those molecules in Fig. 1A, thus generating structural distortions. Although recent works have proved that twisted MR skeletons would benefit the triplet up-conversion process, no one has ever taken the potential spectral broadening and molecular instabilities issues into account (*35*).

Here, we wanted to disclose a tactful strategy to reduce the above steric repulsion by embedding a locking group into MR skeleton, targeting extremely small FWHM and remarkable molecular stability simultaneously. Starting from the carbazole-containing model molecule, BCz-BN, main group elements (C, N, B, O, and S) and hydrogen bond as locking units were theoretically evaluated, and the mesitylboron (BMes) group was found to be an ideal one for a deep blue emitter. Besides planarizing the skeleton by alleviating the repulsions between spatially close hydrogens, this intrinsically stable locking unit enlarges the bond dissociation energy (BDE) of the para-positioned weak carbon-nitrogen (C─N) bond in anionic states, simultaneously narrowing the emission spectra and improving the intrinsic molecular stability. A deep blue emission with a peak at 452 nm and a FWHM of 14 nm/0.09 eV in dilute toluene solution was recorded, achieving CIE coordinates of (0.144, 0.042) that approaches the BT.2020 blue requirement. Using this emitter in a TADF device, an extremely small EL FWHM of 17 nm and a high EQE_max_ of 33.9% can be obtained. A remarkable LT97 of ~180 hours was further recorded in a fluorescence device with a CIE*_y_* of 0.057, nearly 20-fold longer than that of the model emitter (CIE*_y_* of 0.406) and nearly 4-fold times longer than that of a commercial MR emitter (CIE*_y_* of 0.096). Of particular note, nearly all MR emitters feature multiple C─N bonds in skeletons, of which, the stability has been ignored in previous works. The special attentions being paid on stabilizing the molecular stability in this work will surely refresh the molecular design concept and speed up the real applications of MR emitters.

## RESULTS

### Molecular design and theoretical calculation

Our molecular design strategy was illustrated in Fig. 1, starting from the carbazole-embedded sky blue MR emitter, BCz-BN, which actually has been a classical parent skeleton for modification in the literature. Crystal structure of BCz-BN depicts large torsion angles of up to 49.6° between C─H groups of *tert*-butylcarbazole (*t*Cz) segment and the central phenyl ring as well as 31.2° between the C─Hs of two *t*Cz segments, suggesting strong steric repulsions between the spatially close hydrogens (*36*). Those large torsion angles would deteriorate the rigid planar structure of this MR skeleton to induce large structural relaxation on excited states, which should account for the relatively moderate FWHM of 24 nm in toluene. With BCz-BN as the prototype, main group elements (C, N, B, O, and S) and hydrogen bond were introduced to the para position of one nitrogen atom as locking groups. As shown in Fig. 1 (B and C), the simulated emission spectra of BCz-BN and its locked analogs exhibited emission colors across the entire visible spectrum with substantially different FWHMs of 7 to 29 nm. Compared to the parent molecule BCz-BN, the steric hindrance of adjacent C─Hs in the locked-analogs became smaller indicated by the reduced torsion angles. Among them, DBCz-Mes with a BMes group displayed the bluest emission with the narrowest FWHM owing to its smallest reorganization energy (λ) predicted by the Molecular Materials Property Prediction Package(MOMAP) software (*37*, *38*). As shown in fig. S1, the seven molecules displayed similar vibration mode patterns with major intensity differences found in the high-frequency region (1600 to 1700 cm^−1^, highlighted area in fig. S1). Vibration modes with major contributions to reorganization energy were found to be around 1680 cm^−1^ for the seven molecules with varied λs of 21.00 to 135.34 cm^−1^, which were all related to stretching vibrations of the *t*Cz moieties and the central phenyl ring. It is clear that different locking units exhibited distinct impacts on λs, which should be taken account of for the future design of narrowband emitters using the locking strategy. On the basis of the computational results, introducing the BMes motif can avoid the steric repulsion between the spatially close hydrogens to afford a more rigid and planar structure, which was confirmed by single-crystal x-ray diffraction analysis. The crystal structure of DBCz-Mes [The Cambridge Crystallographic Data Centre (CCDC) number: 2209063] revealed small torsion angles of 0.8° between *t*Cz segment and the central phenyl ring and 15.4° between the two *t*Cz segments, which should be advantageous to reduce structural relaxation and narrow emission spectrum. On the other hand, the formed linear B-π-N structure would also reduce the electron-donating ability of the N atom and thus blue-shifts the emission color of this molecule compared with BCz-BN and molecules with other locking groups.

**Fig. 2.**
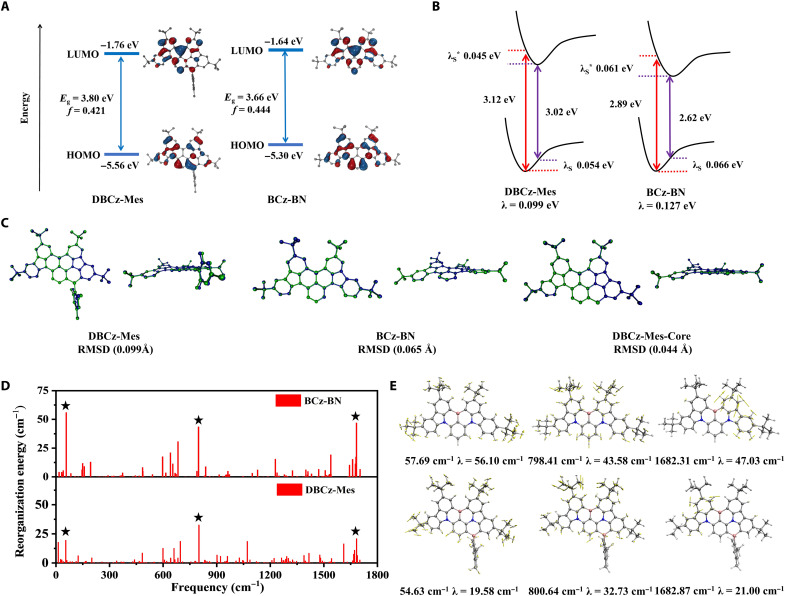
Comparison of BCz-BN and DBCz-Mes. (**A**) HOMO and LUMO distributions, energy band gaps, and oscillator strengths of DBCz-Mes and BCz-BN. (**B**) Reorganization energies of DBCz-Mes and BCz-BN computed at the PBE0/6-31G(d,p) level. (**C**) Top view, side view, and the root mean square deviation (RMSD) values of the optimized structures of DBCz-Mes, BCz-BN, and the core structure of DBCz-Mes in their S_0_ and S_1_ states. (**D**) Transition frequencies and reorganization energies of the vibrational modes contributing to the S_1_-S_0_ transition of DBCz-Mes and BCz-BN. (**E**) Top three vibrational modes for the S_1_-S_0_ transition of DBCz-Mes and BCz-BN; λ, reorganization energy.

To explain the main causes of the narrowed emission of DBCz-Mes, its reorganization energies of the S_1_-S_0_ transition were computed and compared with those of BCz-BN (table S1). As shown in Fig. 2D, two emitters displayed similar vibration mode patterns but substantially smaller reorganization energies for DBCz-Mes, indicating its more suppressed structural distortion during the emission process. The top three vibrational modes involved in the emission of BCz-BN could be assigned to scissoring of two *t*Cz groups (57.69 cm^−1^), scissoring/stretching of the whole molecule (798.41 cm^−1^), and stretching of one *t*Cz group (1682.31 cm^−1^) with λs of 56.10, 43.58, and 47.03 cm^−1^, respectively (Fig. 2E), which are all related with the twisted structure between the *t*Cz segment and the central phenyl ring. The stretching mode at 1682.31 cm^−1^ clearly validated the steric repulsions between adjacent hydrogens, as the two hydrogen atoms on the right *t*Cz group showed large displacement vectors away from the spatially close hydrogens on the left *t*Cz group and the central phenyl ring, respectively. The same three vibrational modes also contributed the most to the emission of DBCz-Mes, with frequencies at 54.63, 800.64, and 1682.87 cm^−1^ but significantly reduced λs of 19.58, 32.73, and 21.00 cm^−1^, respectively. The scissoring mode (~57 cm^−1^) and the stretching mode (~1682 cm^−1^) mainly involved the vibrations of the *t*Cz group; thus, locking it with the central phenyl ring could afford larger decreases in λ (36.52 and 26.03 cm^−1^) compared to that of the scissoring/stretching mode at ~800 cm^−1^ (10.85 cm^−1^). Therefore, theoretical calculation results indicated that the twisted structure caused by the repulsion of spatially close hydrogens in BCz-BN was responsible for the broadened emission spectrum, and our method of BMes locking is one effective solution overcoming it.

### Synthesis and characterization

The synthesis of DBCz-Mes was accomplished in two steps from commercially available starting materials via nucleophilic aromatic substitution reaction with 3,6-di-*tert*-butylcarbazole followed by a “one-pot” lithiation-borylation reaction as we reported recently (fig. S19) (*33*). Two boron atoms were introduced simultaneously via double lithium-halogen exchange, and mesityl Grignard reagent was used to finalize the reaction. The target molecule was air- and moisture-stable and was fully characterized by ^1^H and ^13^C nuclear magnetic resonance (NMR) spectroscopy and mass spectrometry (MS). The thermogravimetric analysis identified the good thermal stability of the emitter with a high decomposition temperature (defined as the temperature at 5% weight loss) of 398°C, and no obvious glass transition signal was found between 75° and 350°C from the differential scanning calorimetry curve, as shown in fig. S2A. The electrochemical behavior of DBCz-Mes was investigated by cyclic voltammetry. As shown in fig. S2B, DBCz-Mes displayed a reduction peak at −2.11 V, which corresponded to a lowest unoccupied molecular orbital (LUMO) level of −2.69 eV. The highest occupied molecular orbital (HOMO) level of DBCz-Mes was then estimated to be −5.43 eV according to its LUMO level and optical bandgap.

**Fig. 3.**
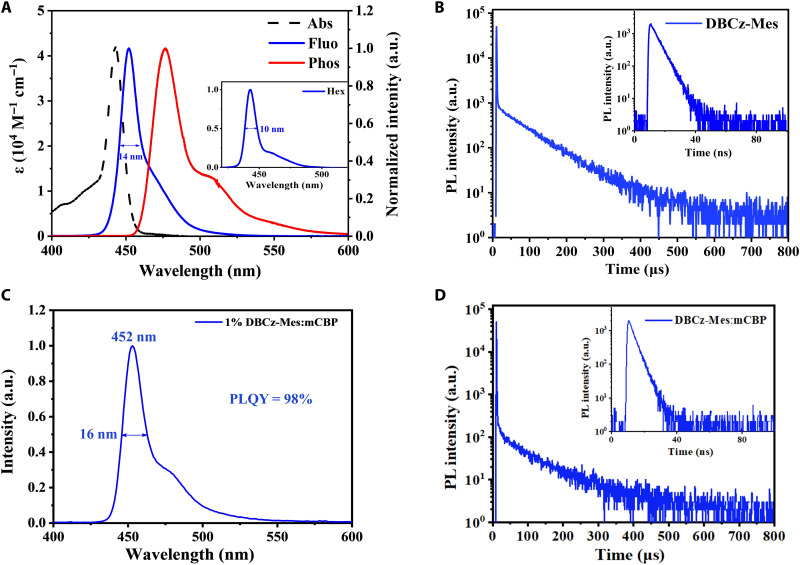
Photophysical characterization. (**A**) Ultraviolet-visible absorption, steady-state PL, and phosphorescence spectra of DBCz-Mes in toluene (10^−5^ M). (**B**) Transient PL decay profiles of DBCz-Mes measured in degassed toluene solution. (**C**) PL spectrum and (**D**) transient PL decay curves of DBCz-Mes in mCBP films at 1 wt % doping level. NTSC, National Television System Committee.

The absorption and emission spectra of DBCz-Mes were measured in toluene (10^−5^ M) as illustrated in Fig. 3A and Table 1. The sharp and intense absorption band at 443 nm was assigned as the short-range CT (SRCT) transition, which is typical for MR molecules. In addition, narrowband deep blue emission peaking at 452 nm with a notably small FWHM of 14 nm (89 meV) was recorded for DBCz-Mes, which was considerably blue-shifted and narrower compared to that of BCz-BN (483 nm, 24 nm/123 meV). The extremely small Stokes shift of 9 nm and an FWHM of 14 nm implied that our strategy of introducing the BMes group successfully suppressed structural changes between the ground state and the excited state, thus leading to ultrapure blue emission. Corresponding CIE coordinates of (0.144, 0.042) was obtained, satisfying the CIE*_y_* requirement of 0.046 for the blue light defined by BT.2020. The photoluminescence (PL) spectra of both DBCz-Mes and BCz-BN in different solvents were also measured as provided in fig. S3 and table S2, revealing positive solvatochromism of 14 and 16 nm, respectively. These behaviors experimentally confirmed the SRCT nature of DBCz-Mes. Impressively, in nonpolar solvent like *n*-hexane, the emission peak of DBCz-Mes further blue-shifted to 443 nm and the FWHM decreased to only 10 nm (63 meV), which was even narrower than most blue-emitting quantum dots (*39*–*41*). As a comparison, BCz-BN only displayed blue-shifted emission at 471 nm and FWHM of 18 nm when dissolved in *n*-hexane.

**Table 1.** Physical properties of DBCz-Mes.

Compound	λ_abs_* (nm)	λ_em_* (nm)	Stokes shift* (nm, meV)	FWHM* (nm, meV)	CIE* (*x*, *y*)	Φ_PL_* (%)	τ_p_* (ns)	τ_d_* (μs)
DBCz-Mes	443	452	9, 56	14, 89	(0.144, 0.042)	99	4.7	80

*Measured in toluene solution (10^−5^ M) at 300 K.

The phosphorescent emission of DBCz-Mes was also measured in the same solution, and the S_1_ and T_1_ energies of DBCz-Mes were deduced from the fluorescence and phosphorescence peaks to be 2.74 and 2.60 eV, respectively, thus giving birth to singlet-triplet energy gap (Δ*E*_ST_) of 0.14 eV, which was sufficiently small to enable TADF. The PL decay curve of DBCz-Mes depicted a clear prompt lifetime of 4.7 ns and a delayed lifetime of 80 μs. Combining with the total photoluminescence quantum yield (PLQY) of ~99% and the portion of the delayed part (Φ_d_ = 58%), a radiative decay rate (*k*_r_) of 8.8 × 10^7^ s^−1^ and a reverse intersystem crossing rate (*k*_RISC_) of 2.9 × 10^4^ s^−1^ were obtained. The photophysical properties of DBCz-Mes were further studied by being dispersed in a wide-energy-gap host, 3,3′-di(carbazol-9-yl)biphenyl (mCBP), with a doping concentration of 1 wt %. The emission of DBCz-Mes in the doped film displayed maximum at 452 nm with a FWHM of 16 nm as shown in Fig. 3C, finely resembling the results in toluene. The slight spectral broadening was associated with the possible host-guest interactions, which was common for MR emitters (*33*, *42*, *43*). Clear TADF characteristics were also confirmed for the doped film by temperature-dependent transient PL decay measurements (Fig. 3D and fig. S4), featuring a short-lived prompt lifetime of 4.16 ns and a long-lived delay lifetime of 114 μs at room temperature, respectively. Furthermore, a high PLQY of ~98% was obtained, evidencing the efficient radiative decay process.

### Device performances

**Fig. 4.**
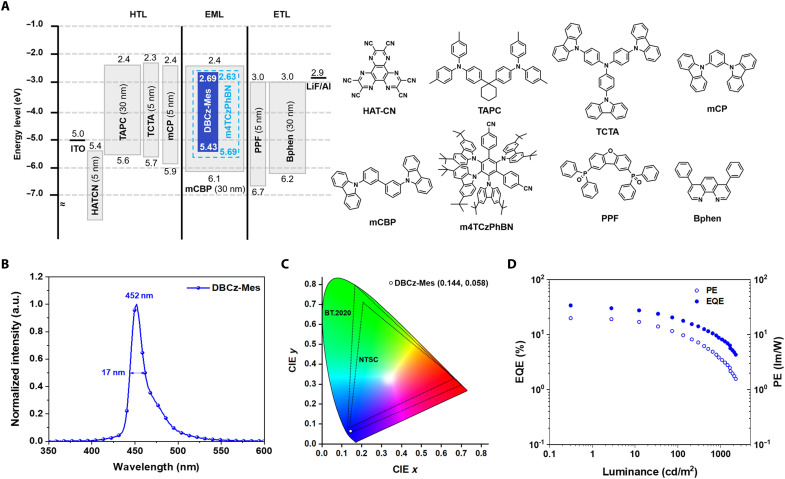
OLED performances of the TSF devices. (**A**) Device configuration, energy level, and molecular structure of the materials used. HTL, hole transporting layer; EML, emitting layer; ETL, electron transporting layer. (**B**) EL spectra of devices recorded at 10 mA/cm^2^. (**C**) CIE color coordinates of the device. (**D**) External quantum efficiency and power efficiency versus luminance curves of the device.

The potential of DBCz-Mes as deep blue emitter in OLED was then evaluated with the device structure of indium-tin-oxide (ITO)/TAPC (30 nm)/TCTA (5 nm)/mCP (5 nm)/mCBP: 30 wt % (2′s,4′r,5′r,6′s)-2′,4′,5′,6′-tetrakis(3,6-di-*tert*-butyl-9H-carbazol-9-yl)-[1,1′,3′,1″-terphenyl]-4,4″-dicarbonitrile (m4TCzPhBN): 1 to 3 wt % DBCz-Mes (30 nm)/PPF (5 nm)/Bphen (30 nm)/LiF (0.5 nm)/Al (150 nm), where TAPC, TCTA, mCP, PPF and Bphen stand for 1,1-bis[4-[*N*,*N′*-di(*p*-tolyl)amino]phenyl]-cyclohexane, 4,4′,4′-tris(carbazol-9-yl)-triphenylamine, 1,3-di-9-carbazolyl-benzene, 2,8-bis(diphenylphosphoryl)-dibenzo[b,d]furan and 4,7-diphenyl-1,10-phenanthroline, respectively. The energy level scheme of the device and the chemical structures of the materials used were depicted in Fig. 4A. Similar to most previously reported works, a TADF sensitizer was used to assist the harvest of triplet excitons to compensate the low *k*_RISC_ of the MR emitter (*44*–*49*). m4TCzPhBN was chosen as the sensitizer because of the high triplet energy level (2.86 eV), fast *k*_RISC_ (~1.0 × 10^6^ s^−1^), and suitable spectral overlap with the absorption peak of DBCz-Mes (fig. S5 and table S3) (*50*). The delayed lifetimes (τ_d_s) of the mCBP:m4TCzPhBN:DBCz-Mes and mCBP:m4TCzPhBN films were measured to be 1.8 and 6.0 μs, respectively, as shown in table S3. The reduced ratio of delayed emission and substantially decreased τ_d_ after introducing the MR emitter clearly indicated efficient Förster energy transfer (FET) process from the sensitizer to the dopant, which was supported by a large FET radius (*R*_0_) of 3.58 Å. In addition, a high horizontal dipole ratio (Θ_//_) of 83% was determined for the DBCz-Mes–doped film (fig. S6), which would enhance outcoupling efficiency.

As illustrated in Fig. 4B, the EL spectrum of the device with 1% DBCz-Mes exhibited deep blue emission peaking at 452 nm with an extremely narrow FWHM of 17 nm, which was consistent with the PL spectrum of the ternary-doped film. To the best of our knowledge, only few MR emitters can realize FWHMs of less than 20 nm in OLEDs, regardless of their colors (*17*, *24*, *25*, *46*–*49*). Our device here represented one of the narrowest EL spectrum in literature, validating the effectiveness of this locking strategy. Notably, the corresponding CIE coordinates reached (0.144, 0.058), which satisfies the requirement of blue light (0.14, 0.08) defined by the National Television System Committee and is close to the BT.2020 standard, representing one of the purest blue colors among boron-based MR emitters (fig. S7 and table S4) (*51*–*56*). Furthermore, as shown in Table 2, the device displayed impressive EL performances, including low turn-on voltage (*V*_on_) of 3.1 V, a high EQE_max_ of 33.9%, and a high power efficiency (PE_max_) of 19.9 lm/W, respectively, which were among the best results of previously reported deep blue OLEDs based on MR emitters. Note that, despite the slightly dropped efficiencies, the EL spectra and color purity of the devices were well maintained as the doping concentration increased from 1 to 3% (fig. S8 and table S5). A plausible reason is the steric hindrance provided by the pedant mesityl group suppresses intermolecular π-π interaction–induced spectral broadening. It should also be mentioned that a large efficiency roll-off was observed with EQE of 8.5% at 1000 cd/m^2^. This is a common situation for other reported deep blue emitters in literature as deep blue emitters always require wide-energy-gap host and other functional transporting materials, which makes it hard to balance charges in devices. Moreover, we believe that the performance of DBCz-Mes under high luminance was also limited by the sensitizer adopted, and a TADF compound with a bluer emission, a higher solid state PLQY, and a faster *k*_RISC_ than m4TCzPhBN may resolve this issue, which, however, was unobtainable at this moment. The OLED devices without sensitizer were also fabricated for comparison, and the data were listed in fig. S9 and table S6. The sensitizer-free devices based on DBCz-Mes showed a reduced EQE_max_ of 16.2% and more significant efficiency roll-offs. The EL spectra of those devices showed high color purity with extremely small FWHMs of merely 16 nm, emission peaks at 451 nm, and CIE*_y_* of ~0.04. The narrowed emission spectra should arise from the reduced polarity of mCBP only, as the spectra of BN emitters would be always broadened in high polarity host due to their SRCT characters.

**Table 2.** EL properties of the OLED devices based on DBCz-Mes.

λ_EL_* (nm)	*V*_on_† (V)	EQE_max_‡ (%)	PE_max_§ (lm/W)	FWHM║ (nm)	CIE¶ (*x*, *y*)
452	3.1	33.9	19.9	17	(0.144, 0.058)

*EL emission maximum, measured at 10 mA/cm^2^.

†Turn-on voltage, measured at 1 cd/m^2^.

‡Maximum external quantum efficiency.

§Maximum power efficiency.

║FWHM of the EL spectrum.

¶Commission Internationale de l’Éclairage coordinates.

**Fig. 5.**
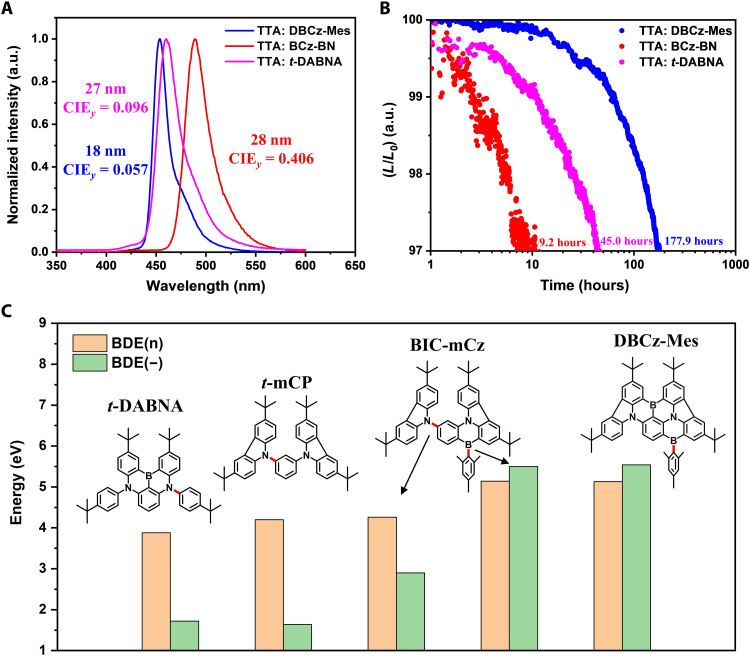
OLED performances of the fluorescent devices. (**A**) EL spectra recorded at 10 mA/cm^2^. (**B**) lifetime data at a constant current density of 12 mA/cm^2^ of the fluorescent OLEDs based on DBCz-Mes and BCz-BN. (**C**) BDE(n) and BDE(−) values of C─X bonds in *t*-DABNA, DBCz-Mes, *t*-mCP, and BIC-mCz.

Despite the high efficiency of above TADF devices, commercialized blue OLEDs still relay on device structures with TTA. To evaluate the intrinsic stability of DBCz-Mes, we further constructed a fluorescent device using DBCz-Mes as the emitter with a stable anthracene derivative as host (fig. S10A). The prototype molecule (BCz-BN) and *t*-DABNA (a benchmark blue MR dopant in fluorescent devices) were also evaluated for comparison. To confirm the TTA mechanism in the fluorescence devices, the PL performance of DBCz-Mes in the TTA host was measured. As shown in fig. S11 and table S7, the doped film [9-(α-naphthyl)-10-(β-naphthyl)-anthracene (α,β-ADN): 1 wt % DBCz-Mes] displayed deep blue emission peaking at 454 nm with a FWHM of 18 nm and a high PLQY of 97%. The transient PL data clearly indicated that the emission of DBCz-Mes doped in α,β-ADN is pure fluorescence with a short lifetime of 3.28 ns. As expected, the delayed component of was DBCz-Mes completely quenched by the TTA host due to its low triplet energy level. The EL transient decay curve of the fluorescence device based on DBCz-Mes was also measured. As illustrated in fig. S12, after a fast prompt decay, a clear delayed EL component was observed, indicating the presence of the TTA process under EL excitation. The delayed emission ratio was estimated to be 32%, leading to the total radiative exciton ratio of about 37%, which agreed well with the previous report (*20*) and further confirmed the involvement of the TTA process (fig. S13). As shown in Fig. 5A and table S8, the device doped with 1% DBCz-Mes exhibited not only clearly blue-shifted and narrowed EL (λ_EL_ = 454 nm, FWHM = 18 nm, and CIE*_y_* = 0.057) but also higher EQE_max_ (10.3%) compared to the ones with 1% BCz-BN (λ_EL_ = 490 nm, FWHM = 28 nm, CIE_y_ = 0.406, and EQE_max_ = 9.0%) and 1% *t*-DABNA (λ_EL_ = 460 nm, FWHM = 27 nm, CIE_y_ = 0.096, and EQE_max_ = 9.2%). Impressively, when measured at a constant current density of 12 mA/cm^2^, the device based on DBCz-Mes manifested an outstanding LT97 of 177.9 hours, which was nearly 20-fold and 4-fold longer than those of the BCz-BN–doped device (9.2 hours) and *t*-DABNA–doped device (45.0 hours) (Fig. 5B). It should be pointed out that, normally, the device operational stability decreases sharply with the blue-shifted EL emission wavelengths, especially for blue OLEDs (*57*). Considering that the improvement in device stability of DBCz-Mes was realized with its much bluer emission compared with that of BCz-BN and *t*-DABNA, the excellent intrinsic stability of our molecule was more intriguing. The rigid and planar structure of DBCz-Mes should account partly for those results, stabilizing the empty p orbital of the boron atom by the more delocalized π electrons and reducing structural deformation upon excitation. Moreover, we also theoretically calculated the BDE(n) and BDE(−) of the C─X bonds (X = N, B) in a series of representative emitters, namely, *t*-DABNA, DBCz-Mes, *t*-mCP, and BIC-mCz, at the M06-2X/def2-SVP level for a deep understanding on the role of this locking group, where the BDE(n) and BDE(−) refer to the BDEs under neutral and anionic states, respectively. Generally, C─X bonds have been recognized as the Achilles’ heels of the molecules as they are particularly vulnerable in negative charged states (*58*–*61*). As shown in Fig. 5C, the calculated BDE(−) value of *t*-DABNA was merely 1.72 eV, which was considerably smaller than its BDE(n) of 3.88 eV. On the other side, the C─B bond in DBCz-Mes was extremely stable, as indicated by high BDE(n) and BDE(−) values of 5.13 and 5.44 eV, respectively. Such difference between BDE(−) of C─N and C─B bonds can be attributed to (i) the intrinsic BDE of C─B bond (393 kcal/mol) is higher than that of the C─N bond (305 kcal/mol) and (ii) the electronegativity of boron atom is lower than that of carbon atom, unlike that of nitrogen atom. As indicated in a recent report, BDE(−) of a C─X bond is heavily affected by the electron affinity of the anion containing segment, which often contains the more electronegative X atom (*61*). For DBCz-Mes, it was the mesityl moiety that contained the negative charge, which resulted in largely reduced electron affinity compared to the carbazole moieties in *t*-DABNA, *t*-mCP, and BIC-mCz (table S9). The BDE(+) of the C─B bond was also calculated to confirm its bond strength in the cationic state. While lower than its own BDE(n) and BDE(−), a BDE(+) of 3.87 eV was comparable to the BDE(n) of the C─N bonds and much higher than their BDEs(−), suggesting high stability of the pendant C─B bond in EL devices. As for now, it was unable to directly calculated the BDE(n) and BDE(−) of the C(Ph)-N(Cz) bond (the one connecting the carbazole and the central benzene) in BCz-BN and DBCz-Mes to compare their bond strengths. As an alternative, one can calculate the BDE(n)s and BDE(−)s of the C(Ph)-N(Cz) bond in *t*-mCP and BIC-mCz to study the effect of the addition of para-positioned BMes group. Albeit almost the same BDE(n)s, the BDE(−)s of the C(Ph)-N(Cz) bonds were found to be much higher in BIC-mCz (2.90 eV) than that in *t*-mCP (1.64 eV). This result clearly indicated that the addition of BMes group could greatly stabilize the para-positioned C─N bond of the molecule under anionic state and supported our experimental results. Furthermore, as shown in table S10, our device performances are among the best reported so far for blue fluorescent OLEDs with boron-derived MR emitters and recent reported solid-solution crystalline devices in terms of efficiency, color purity, and lifetime, which could guide subsequent designs of stable narrowband blue emitters toward ultrahigh-definition OLEDs (*62*). What should be further pointed out is that C─N bonds exist in most organic semiconductors, including MR emitters. In addition, plenty of works have devoted into stabilizing C─N bonds particularly in anionic states to prevent the intrinsic degradation of molecules (*60*). However, no one has ever considered this issue in MR compounds, particularly the C─N bonds in MR skeletons. Our work here not only deepens the understanding of the influence of C─N bond on stability of MR emitters but also provides a universal strategy to stabilize it, which will surely motivate the design concept of MR emitters that integrating both color purity and stability for practical applications.

## DISCUSSION

In summary, we presented a locking strategy for blue-shifting and narrowing the emission of MR-TADF molecules and simultaneously enhancing their chemical stability. By locking the central phenyl ring and one *t*Cz group of the parent compound BCz-BN with the BMes group, we achieved (i) blue-shifted emission due to the N-π-B unit, (ii) narrowed emission FWHM owing to the reduced structural relaxation at excited states by the highly planar MR structure, and (iii) improved chemical stability indicated by the large increase of BDE(−) value of the C(Ph)-N(Cz) bond. The corresponding MR emitter DBCz-Mes displayed deep blue emission at 452 nm with a notably small FWHM of 14 nm in dilute toluene solution, which could further decrease to 10 nm in dilute *n*-hexane solution. OLED device using DBCz-Mes as the emitter displayed impressive EL performances with an ultrapure blue emission peaking at 452 nm, a small FWHM of 17 nm, and an EQE_max_ of 33.9%. Furthermore, fluorescent OLEDs based on DBCz-Mes exhibited an around 20 and 4 times longer lifetime than those of the BCz-BN– and *t*-DABNA–doped devices, respectively, breaking the general trend that the device operational stability decreases as emission wavelength becomes shorter and revealing the advantages of this BMes locking strategy in simultaneously blue-shifting the emission and improving molecular stability. Those state-of-the-art performances greatly validated the effectiveness of the design strategy here. Besides the awareness of the crucial importance of alleviating the steric repulsions in MR molecules, those findings in this work will urge research studies to pay more attention on the stability issues of MR emitters, lastly paving the way toward highly efficient and stable narrowband BT.2020 blue emitters.

## MATERIALS AND METHODS

### General

All reactions were carried out under a N_2_ atmosphere using Schlenk techniques unless otherwise noted. Reagents were purchased from commercial sources and used as received. ^1^H and ^13^C NMR spectra were recorded on a JEOL JNM-ECS600 spectrometer at room temperature in deuterated chloroform. Matrix-assisted laser desorption/ionization–time-of-flight (MALDI-TOF) MS data were performed on a Shimadzu AXIMA Performance MALDI-TOF instrument in positive detection modes. The energy levels of DBCz-Mes were measured by cyclic voltammetry using a CHI600E electrochemical workstation. The Pt/C was used as working electrode, platinum wire as auxiliary electrode, and an Ag/AgCl system as reference electrode standardized against ferrocene/ferrocenium. *N*,*N*-dimethylformamide was used for the reduction potentials. The concentrations were 5 mg/ml for all compound, and the scan rate of 100 mV s^−1^ was adopted. Single crystals of DBCz-Mes were grown from CH_2_Cl_2_ by slow evaporation of the solvent. A suitable crystal was selected and mounted on glass fibers, and the data were collected on a XtaLAB Synergy R, DW system, HyPix diffractometer. The crystal was kept at 179.99 (10) K during data collection. Using Olex2, the structure was solved with the ShelXT structure solution program using intrinsic phasing and refined with the ShelXL refinement package using least squares minimization (table S11) (*63*–*65*).

### FET radii calculation

The FET radii (*R*_0_) is defined as the intermolecular distance at which the energy transfer rate constant is equal to the total decay rate constant of the donor in absence of the acceptor. Correspondingly, *R*_0_ can be expressed in the following equation (*33*)

$$R_{0}^{6}=\phi_{D}k^{2}\left[\frac{9000(\ln10)}{128\pi^{5}N_{A}n^{4}}\right]\int_{0}^{\infty }F_{D}(\lambda )\varepsilon_{A}(\lambda )\lambda^{4}d\lambda$$

where ϕ_D_ is the fluorescence quantum yield of the donor in the absence of acceptor, *k*^2^ is a configurational factor describing the relative orientation of transition dipoles of the donor and acceptor, *N*_A_ is Avogadro’s number, *n* is the refractive index of the medium, *F*_D_(λ) is the normalized emission spectra of the donor, and ε_A_(λ) is the molar absorption coefficient of the acceptor. For randomly distributed donor-acceptor system, *k*^2^ is usually assumed to be 2 of 3, while, for most of the organic materials, *n* is about 1.7.

### Theoretical calculations

Theoretical calculations were carried out using the Gaussian 16 software (Rev. B01) (*66*). The ground-state geometries of all molecules and time-dependent density functional theory (TD-DFT) calculations were performed at the PBE0/6-31G(d,p) level (*67*). The root mean square deviations of the optimized structures at S_0_ and S_1_ states were analyzed by Visual Molecular Dynamics (VMD) software (*68*). Fluorescence spectra were simulated using the MOMAP software.

### Photophysical measurement

Solutions (1 × 10^−5^ M) were prepared by stepwise dilution for solution measurements. Thin films for photophysical characterization were prepared by spin coating of the corresponding solid samples dissolved in tetrahydrofuran at the concentration of 10 mg/ml. Ultraviolet-visible absorption and PL spectra were measured using UV-2600 (Shimadzu) and FluoroMax-4P (Horiba) instruments. Fluorescent and phosphorescent spectra were recorded at 298 and 77 K, respectively, at an excitation wavelength of 360 nm. The PLQYs were obtained with an absolute PL quantum yield measurement system Hamamatsu C9920-03G in an integrating sphere. The solution samples were bubbled with nitrogen for 10 min before measurement, while the films were measured in air. The transient spectra were collected on an Edinburgh Fluorescence Spectroscopy FLS1000.

### Device fabrication and measurement of EL characteristics

All compounds were subjected to temperature-gradient sublimation under high vacuum before use. OLEDs were fabricated on the ITO-coated glass substrates with multiple organic layers sandwiched between the transparent-bottom ITO anode and the top metal cathode. Before device fabrication, the ITO glass substrates were precleaned carefully. All material layers were deposited by vacuum evaporation in a vacuum chamber with a base pressure of 10^−6^ torr. The deposition system permits the fabrication of the complete device structure in a single vacuum pump-down without breaking vacuum. The deposition rate of organic layers was kept at 1 to 2 Å/s. The doping was conducted by coevaporation from separate evaporation sources with different evaporation rates. The current density, voltage, luminance, external quantum efficiency, EL spectra, and other characteristics were measured with a Keithley 2400 source meter and an absolute EQE measurement system in an integrating sphere at the same time. The EQE measurement system is Hamamatsu C9920-12, which equipped with Hamamatsu PMA-12 Photonic multichannel analyzer C10027-02 whose longest detection wavelength is 1100 nm. Device encapsulation was carried out in glove box for following performance characterizations and lifetime measurements. Device lifetimes were measured using an OLED aging lifetime tester (ZJZCL-1, Shanghai University).

## Supplementary Materials

### This PDF file includes:

Synthesis and Characterization Figs. S1 to S19 Tables S1 to S11 References
