# Supplementary material for: Improving the stability and color purity of a BT.2020 blue multiresonance emitter by alleviating hydrogen repulsion
Source: Sci Adv. 2023 May 12;9(19):eadh1434. doi: 10.1126/sciadv.adh1434 (PMC10181185; doi:10.1126/sciadv.adh1434)
Supplement: Supplementary file 1 — Synthesis and Characterization Figs. S1 to S19 Tables S1 to S11 References [file sciadv.adh1434_sm.pdf]

Science Advances
AAAS

Supplementary Materials for

Improving the stability and color purity of a BT.2020 blue multiresonance emitter by alleviating hydrogen repulsion

Xiang Wang *et al.*

Corresponding author: Dongdong Zhang, ddzhang@mail.tsinghua.edu.cn; Lian Duan, duanl@mail.tsinghua.edu.cn

Sci. Adv. **9**, eadh1434 (2023)
DOI: 10.1126/sciadv.adh1434

This PDF file includes:

Synthesis and Characterization
Figs. S1 to S19
Tables S1 to S11
References

Synthesis and Characterization

Synthesis of 9,9'-(2,4-dibromo-1,3-phenylene)bis(3,6-di-tert-butyl-9H-carbazole)

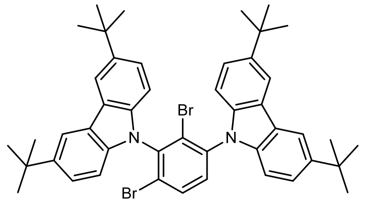

Cs_2CO_3 (9.59 g, 29.42 mmol), 3,6-di-*tert*-butyl-9H-carbazole (4.93 g, 17.65 mmol), 3-dibromo-2,4-difluorobenzene (2.0 g, 7.36 mmol) and 60 ml dry dimethylformamide were added to a 150 ml 3-necked round bottom flask under N_2. The reaction mixtures were stirred at 140 °C overnight. After cooling to room temperature, 300 ml water was added and the precipitates were collected via vacuum filtration. The crude product was dissolved in dichloromethane and washed with water and brine. The organic layer was dried over $MgSO_4$, filtered and the solvent was removed under reduced pressure. The product was purified using flash chromatography through silica (20:1 petroleum ether:CH_2Cl_2 as eluent) to afford 5.01 g 9,9'-(2,4-dibromo-1,3-phenylene)bis(3,6-di-tert-butyl-9H-carbazole) as a white solid (86 % yield). ^{1}H NMR (600 MHz, Chloroform-d) δ 8.16 (dd, J = 14.8, 1.8 Hz, 4H), 7.97 (d, J = 8.5 Hz, 1H), 7.74 – 7.37 (m, 5H), 7.09 (d, J = 8.5 Hz, 2H), 7.01 (d, J = 8.3 Hz, 2H), 1.47 (d, J = 5.0 Hz, 36H). ^{13}C NMR (151 MHz, Chloroform-d) δ 143.44, 143.28, 139.05, 138.79, 138.35, 137.99, 133.39, 132.07, 128.65, 126.18, 124.04, 123.99, 123.55, 123.38, 116.73, 116.61, 109.38, 109.01, 34.88, 32.13, 32.10.

Synthesis of DBCz-Mes

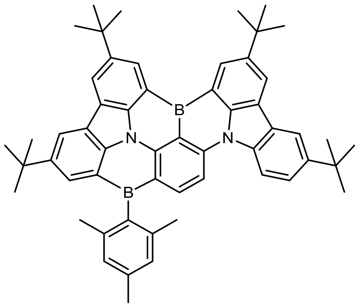

9,9'-(2,4-dibromo-1,3-phenylene)bis(3,6-di-tert-butyl-9H-carbazole) (2.0 g, 2.53 mmol) and 100 ml dry *o*-xylene were added to a 250 ml 3-necked round bottom flask under N_2. The solution was cooled to 0 °C before *n*-butyllithium (2.4 ml, 2.50 M, 6.00 mmol) in hexane was added and the mixture was stirred for 2 h at room temperature. After addition of BBr_3 (1.50 g, 6.0 mmol) at -40 °C, the reaction mixture were stirred at room temperature for 1 h. N,N-Diisopropylethylamine (1.3 ml, 7.5 mmol) was added at 0 °C and the reaction mixture were heated at 120 °C overnight. After cooling to room temperature, mesitylmagnesium bromide (12 ml, 1.0 M, 12.0 mmol) in

tetrahydrofuran was added and the reaction mixture was stirred at room temperature for 12 h. The solvent was removed under reduced pressure and the crude product was dissolved in dichloromethane and washed with water and brine. The organic layer was dried over $MgSO_4$, filtered and the solvent was removed under reduced pressure. The product was purified using flash chromatography through silica (10:1 petroleum ether:CH_2Cl_2 as eluent) to afford 0.44 g DBCz-Mes as a yellow solid (23 % yield). ^1H NMR (400 MHz, Chloroform-d) δ 9.29 (dd, J = 19.6, 1.8 Hz, 2H), 8.68 (dd, J = 7.2, 1.7 Hz, 2H), 8.58 – 8.49 (m, 3H), 8.46 (d, J = 8.8 Hz, 1H), 8.30 (d, J = 2.1 Hz, 1H), 8.19 (d, J = 2.0 Hz, 1H), 7.68 (dd, J = 8.7, 2.1 Hz, 1H), 7.06 (s, 2H), 2.49 (s, 3H), 2.15 (s, 6H), 1.73 (d, J = 5.4 Hz, 18H), 1.55 (d, J = 4.6 Hz, 18H). ^{13}C NMR (101 MHz, Chloroform-d) δ 147.95, 146.54, 146.40, 146.24, 145.63, 143.88, 142.53, 141.43, 140.99, 140.08, 139.68, 138.70, 136.77, 130.95, 130.00, 129.74, 127.87, 127.22, 125.13, 124.94, 124.72, 123.40, 122.83, 121.16, 117.58, 114.98, 108.85, 35.70, 35.62, 35.41, 35.01, 32.46, 32.44, 32.31, 31.93, 23.93, 21.55. MS (MALDI-TOF MS) calculated for $C_{64}H_{70}B_2N_2$ $[M]^+$: 768.4786; found 768.5607.

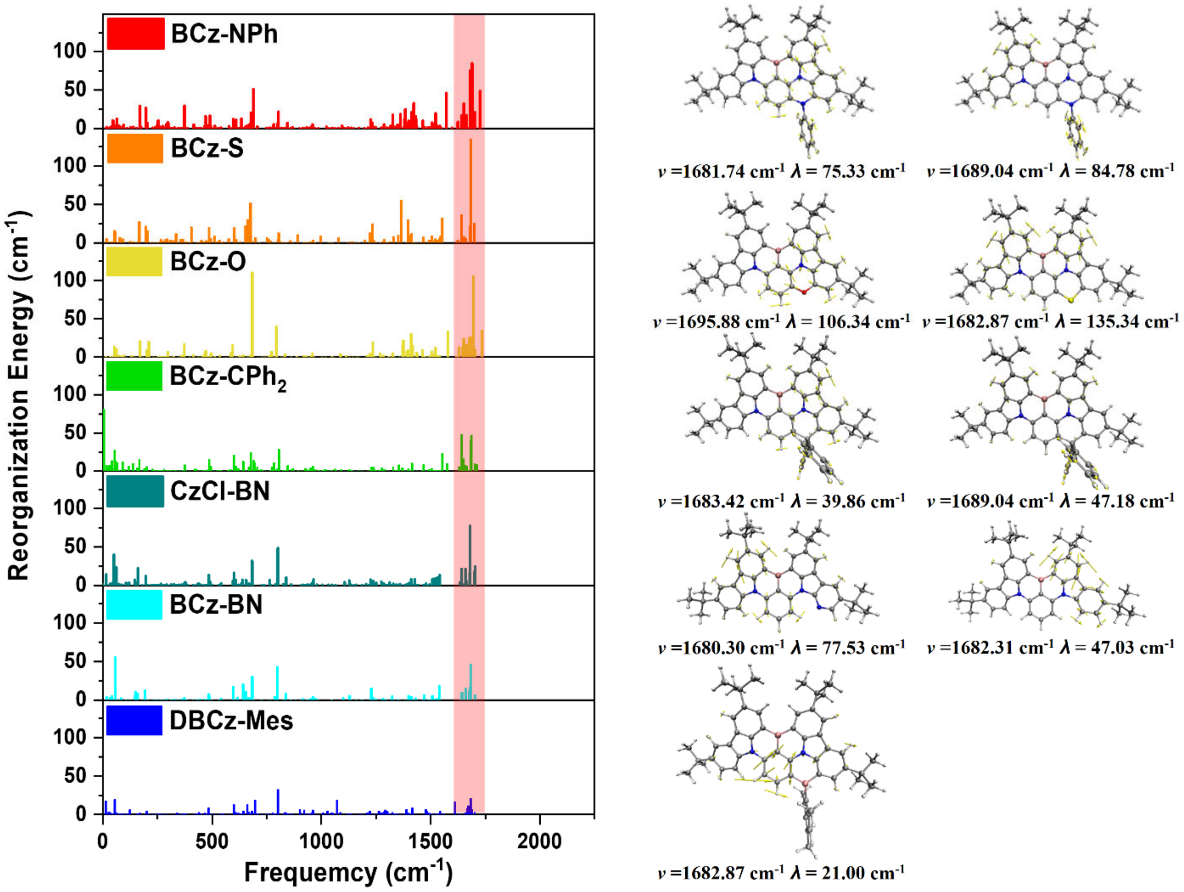

Fig. S1. Additional computational results. Reorganization energy versus frequency plots and dominating vibration modes of DBCz-Mes, BCz-BN, CzCl-BN, BCz-CPh2, BCz-O, BCz-S and BCz-NPh. "ν" and "λ" denote frequency and reorganization energy, respectively.

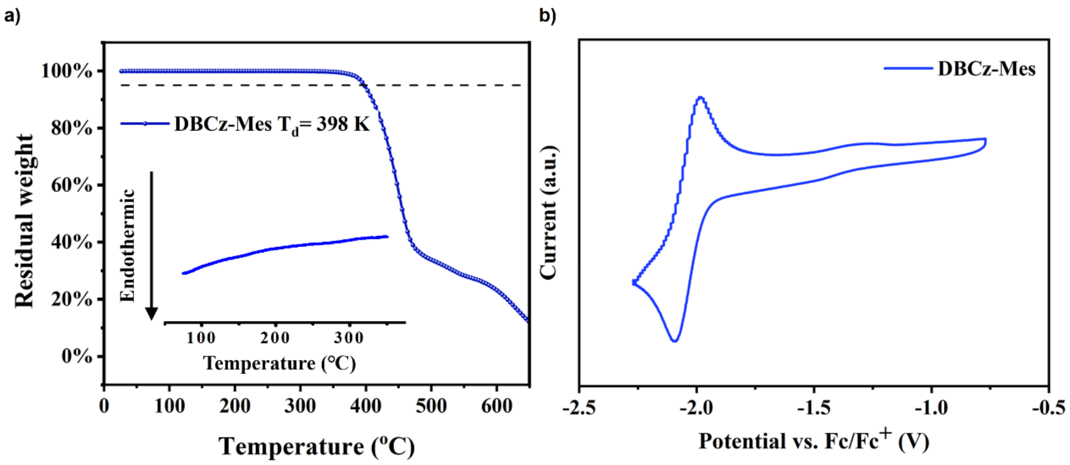

Fig. S2. Thermal and electrochemical properties of DBCz-Mes. (a) TGA curve (inset: DSC trace) and (b) reduction behavior of DBCz-Mes.

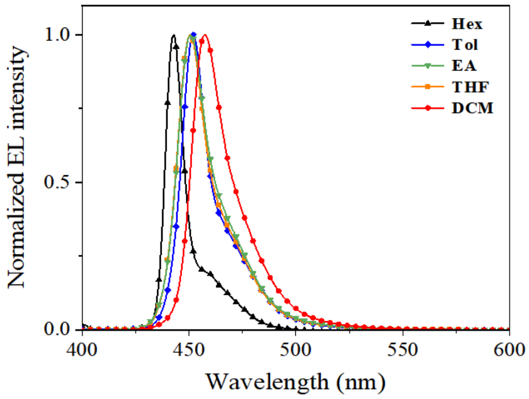

Fig. S3. Solvatochromism property of DBCz-Mes and BCz-BN. Fluorescent spectra of DBCz-Mes (left) and BCz-BN (right) in solvents with different polarities.

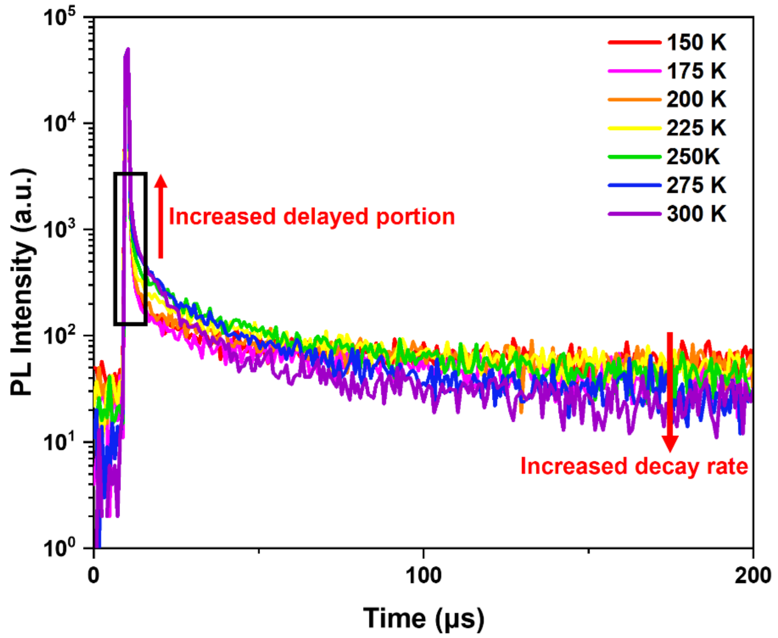

Fig. S4. Temperature-dependent transient PL decay curves of DBCz-Mes in mCBP films at 1wt% doping level.

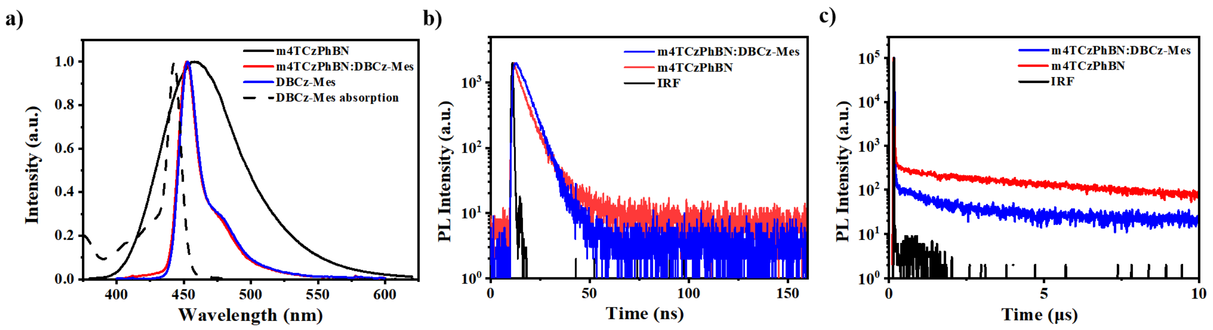

Fig. S5. PL spectra and transient decay curves of DBCz-Mes and the sensitizer in mCBP films. a) The absorption of DBCz-Mes in toluene solution and the PL spectra of the doped films, b) prompt and c) delayed PL decay curves of the mixed films of mCBP: 30 wt% m4TCzPhBN: 1 wt% DBCz-Mes and mCBP: 30 wt%m4TCzPhBN.

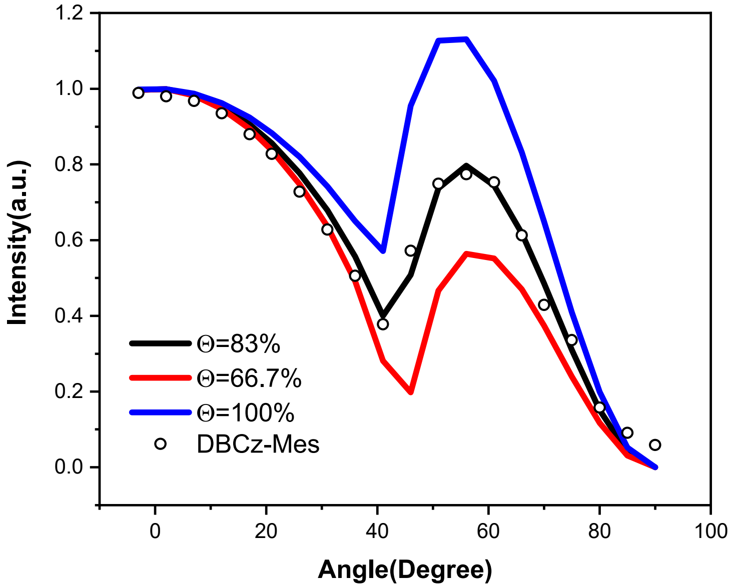

Fig. S6. Angle-dependent PL spectra of DBCz-Mes in mCBP film at 1wt% doping level. The experimental data are in comparison with the fitting curve for different horizontal dipole ratios.

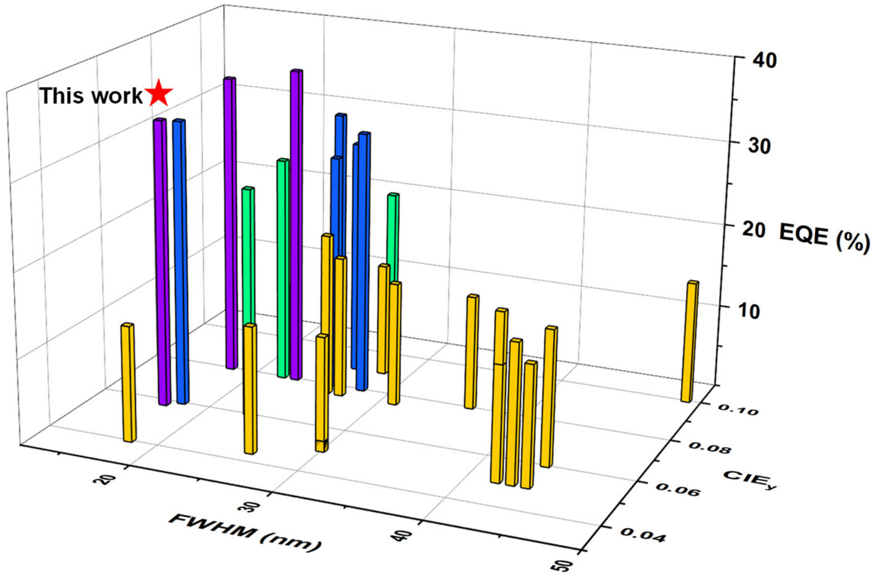

Fig. S7. Summary of OLED performances. EQE vs. CIE_y vs. FWHM plot of DBCz-Mes and other deep-blue boron-based MR emitters in the literature.

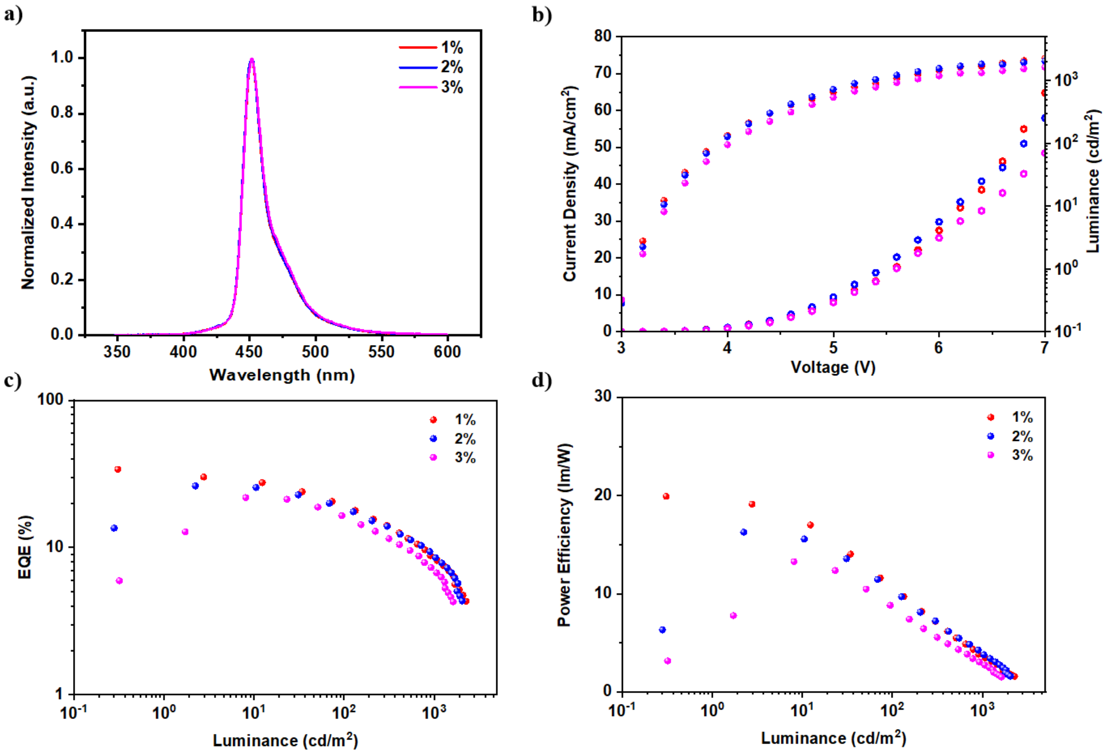

Fig. S8. OLED performances of the sensitizer-type devices. a) EL spectra recorded at 10 mA/cm^2. b) Current density-voltage-luminance (*J*-*V*-*L*) characteristics, c) external quantum efficiency and d) power versus luminance curves of the devices at different DBCz-Mes doping concentrations.

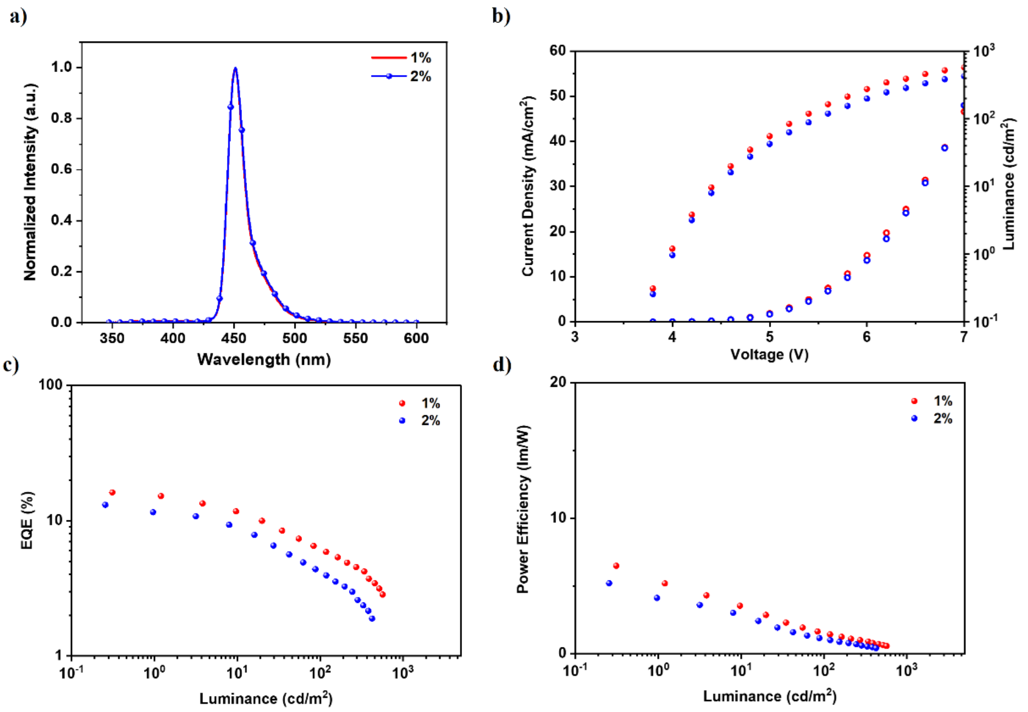

Fig. S9. OLED performances of the sensitizer-free devices. a) EL spectra recorded at 10 mA/cm^2. b) Current density-voltage-luminance (*J*-*V*-*L*) characteristics, c) external quantum efficiency and d) power versus luminance curves of the devices without sensitizer.

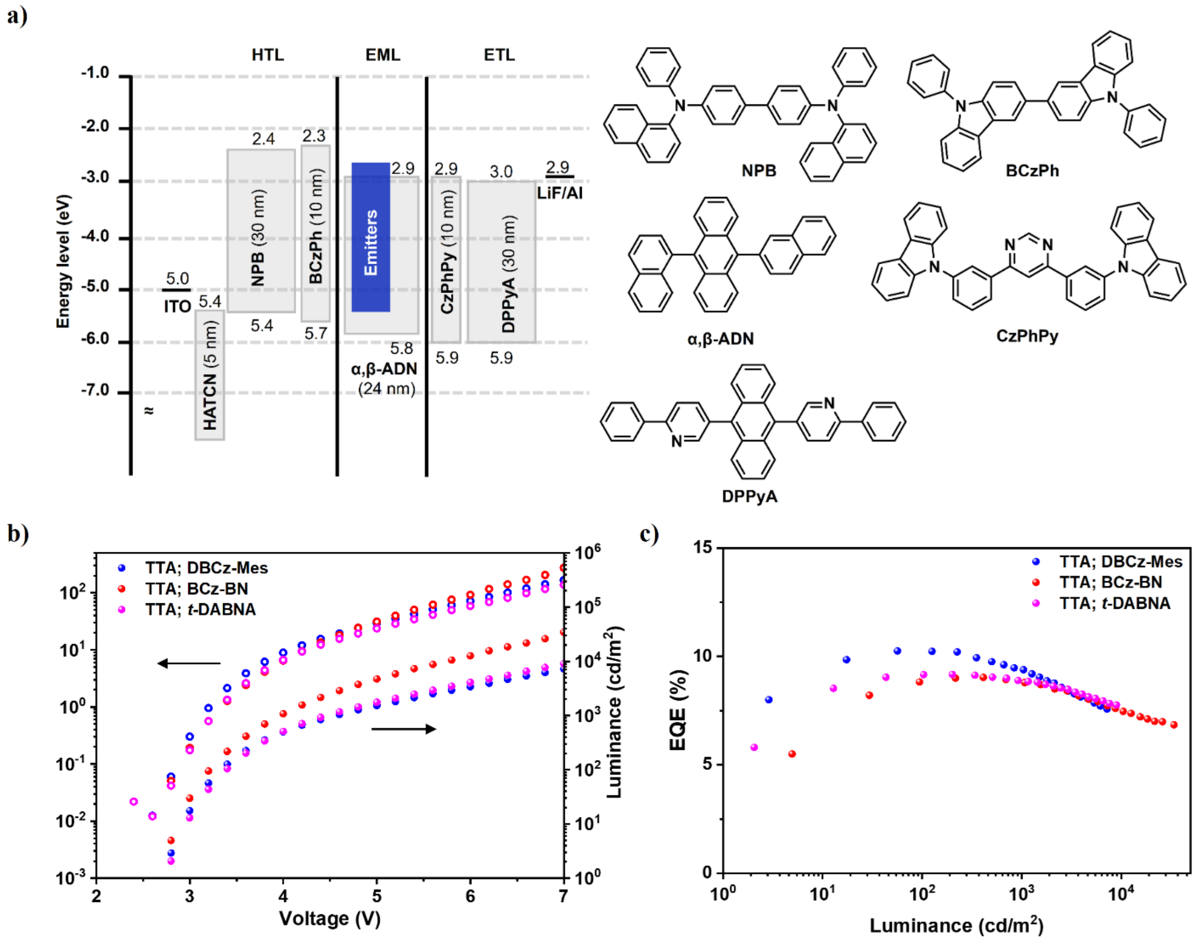

Fig. S10. OLED performances of the fluorescent devices. a) Energy level and molecular structure of the fluorescent devices. b) Current density-voltage-luminance (*J*-*V*-*L*) characteristics, c) external quantum efficiency versus luminance curves.

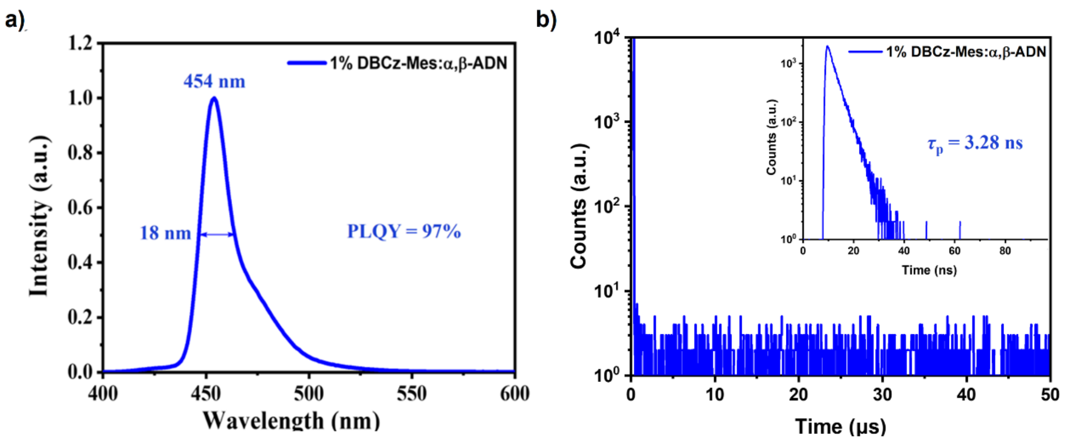

Fig. S11. PL spectra and transient decay curves of DBCz-Mes in α,β-ADN film. a) PL spectrum and b) transient PL decay curves of DBCz-Mes in α,β-ADN film at 1 wt% doping level.

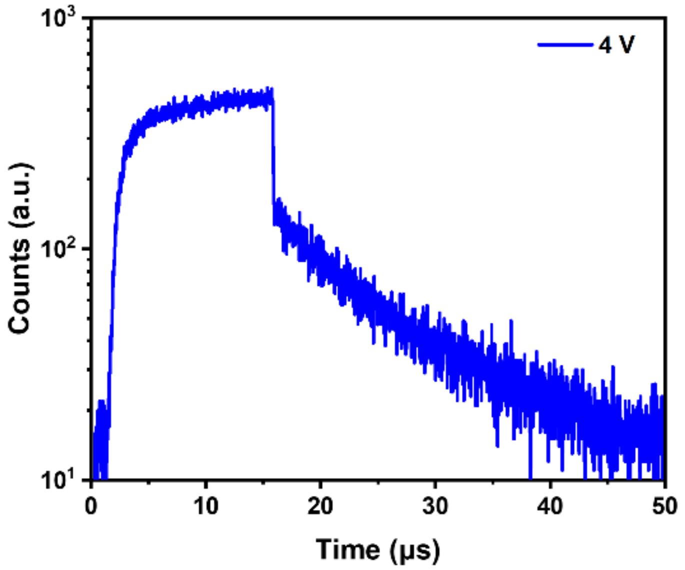

Fig. S12. Transient EL decay curve of the fluorescence device measured at 4 V.

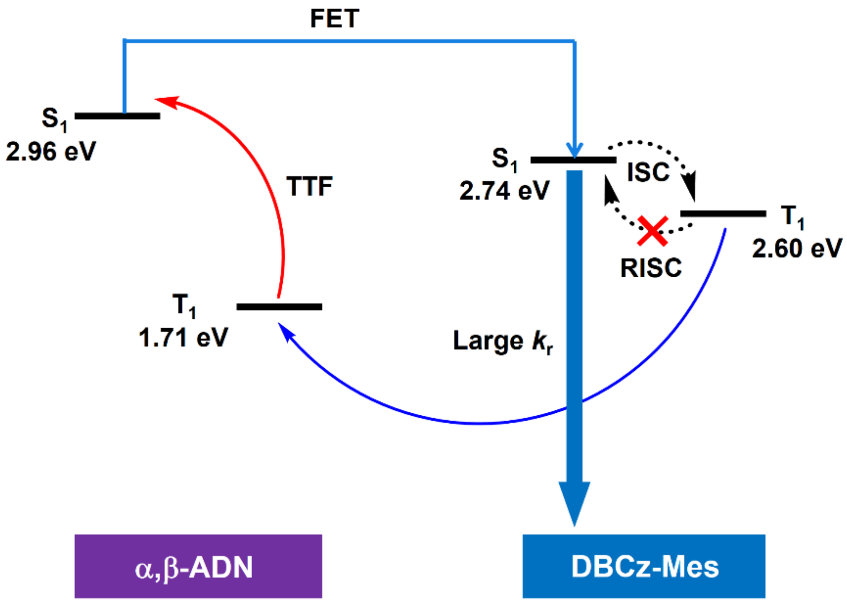

Fig. S13. The energy transfer process in the fluorescence device.

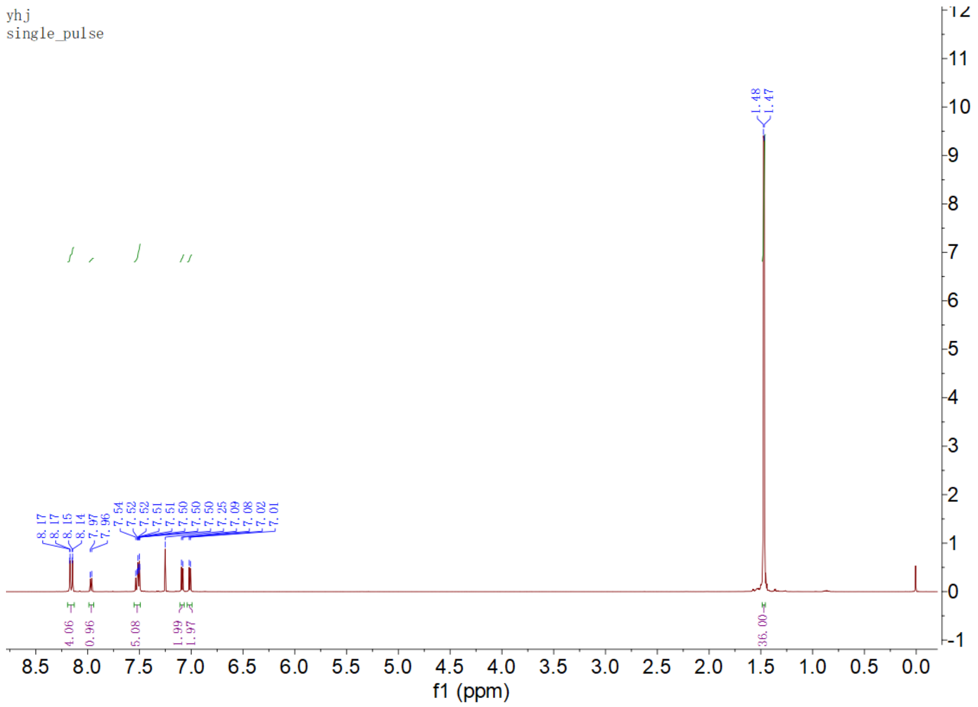

Fig. S14. ^{1}H NMR spectrum of 9,9'-(2,4-dibromo-1,3-phenylene)bis(3,6-di-*tert*-butyl-9H-carbazole) (600MHz, $CDCl_3$).

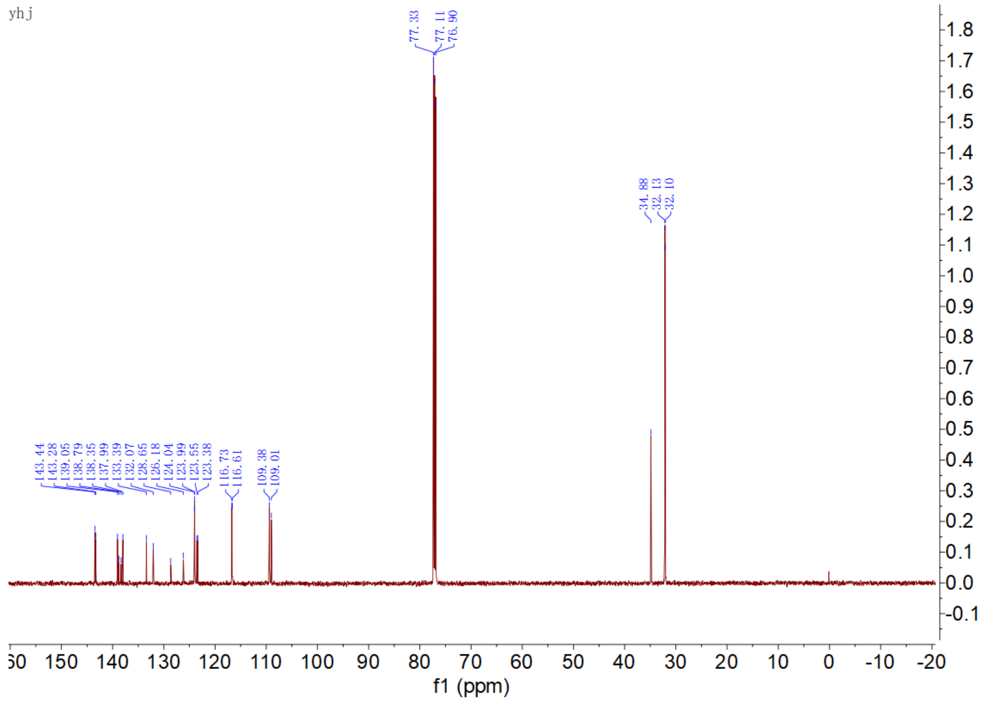

Fig. S15. ^{13}C NMR spectrum of 9,9'-(2,4-dibromo-1,3-phenylene)bis(3,6-di-*tert*-butyl-9H-carbazole) (151MHz, $CDCl_3$).

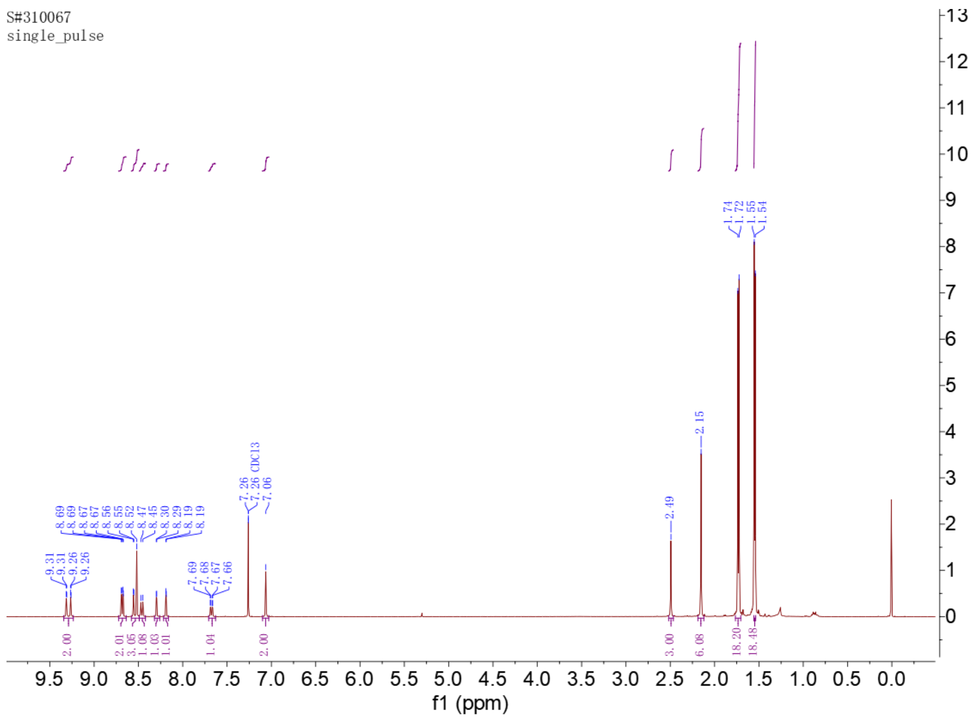

Fig. S16. ^{1}H NMR spectrum of DBCz-Mes (400 MHz, $CDCl_3$).

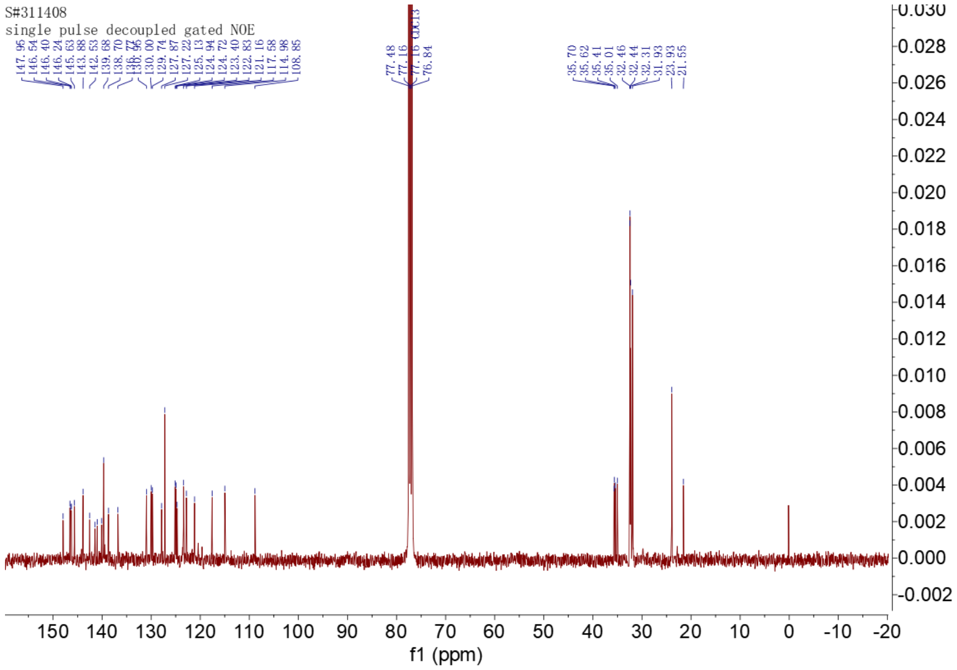

Fig. S17. ^{13}C NMR spectrum of DBCz-Mes (101 MHz, $CDCl_3$).

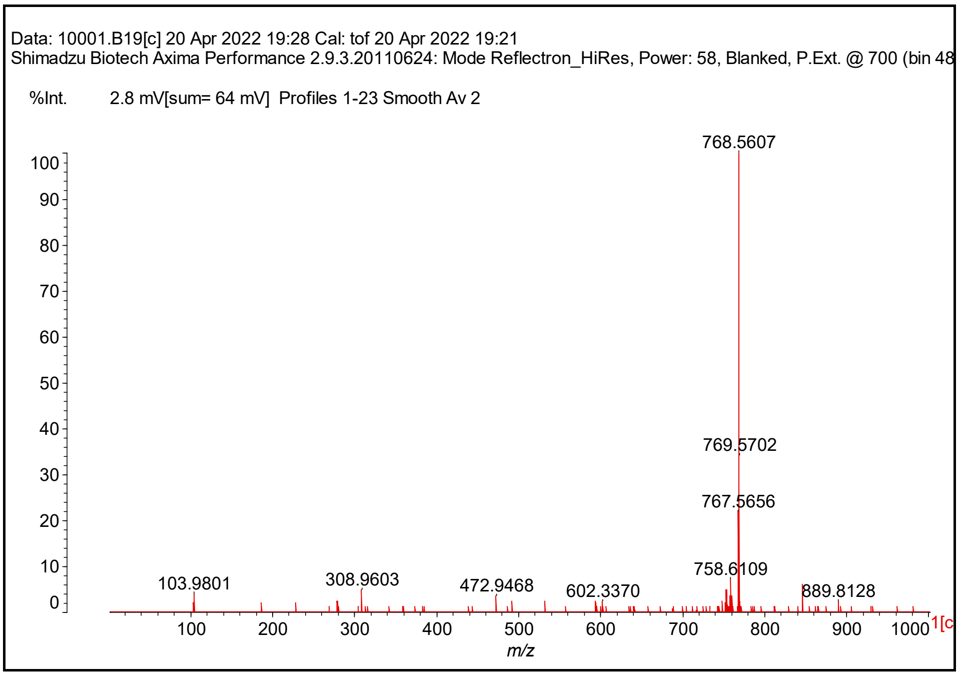

Fig. S18. MALDI-MS spectrum of DBCz-Mes

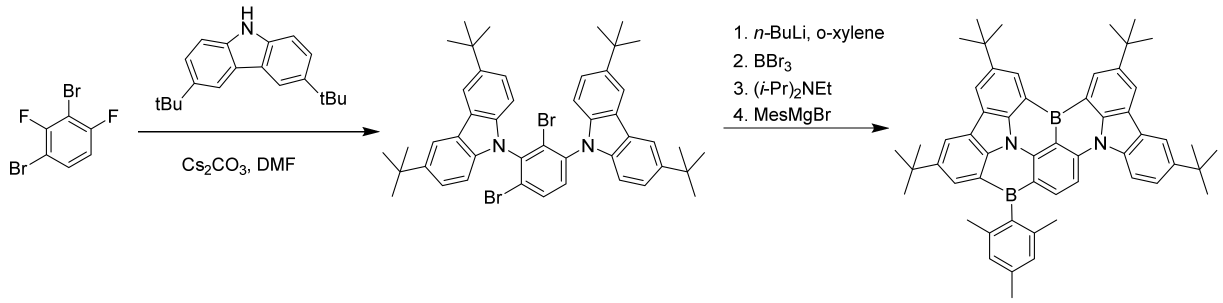

Fig. S19. Synthetic scheme for DBCz-Mes

Supplementary Table

Table S1. Summary of the top five transitional modes contributing to the S_1-S_0 transition of DBCz-Mes and BCz-BN analyzed using the MOMAP software.

Compound	Transition mode	Frequency (cm^{-1})	Huang-Rhys factor	Reorganization energy (cm^{-1})
DBCz-Mes	12	54.63	0.35845	19.58
	109	696.48	0.02692	18.75
	119	800.64	0.04088	32.73
	174	1071.64	0.01748	18.73
	284	1682.87	0.01248	21.00
BCz-BN	9	57.69	0.97233	56.1
	81	641.17	0.03274	20.99
	88	683.16	0.04503	30.76
	98	798.41	0.05458	43.58
	237	1682.31	0.02796	47.03

Table S2. Summary of emission maximum and FWHM (in parentheses) of DBCz-Mes and BCz-BN in solvents with different polarities.

Compd	Hexane (nm)	Toluene (nm)	Tetrahydrofuran (nm)	Ethyl Acetate (nm)	Dichloromethane (nm)
DBCz-Mes	443 (10)	452 (14)	451 (17)	451 (18)	457 (20)
BCz-BN	473 (18)	484 (24)	483 (25)	482 (26)	489 (29)

Table S3. Summary of photophysical data of the doped films.

Composition	λ_{em} (nm)	Φ_{PL} (%)	Φ_p (%)	Φ_d (%)	FWHM (nm)	τ_p (ns)	τ_d (μs)	k_F (10^7 s^{-1})	k_{IC} (10^7 s^{-1})
mCBP:m4TCzPhBN :DBCz-Mes	452	90	72	18	16	5.4	1.8	13.4	1.5
mCBP:m4TCzPhBN	458	48	11	37	70	6.2	6.0	1.8	2.0

Table S4. Summary of reported deep-blue OLEDs based on boron-derived MR emitters with $CIE_y < 0.1$.

Emitter	EQE_{max} (%)	λ_{EL}/FWHM (nm)	CIE $_{(x, y)}$	Ref.
**DBCz-Mes**	**33.9**	**452/17**	**(0.144, 0.058)**	**This work**
DABNA-1	13.5	459/28	(0.13, 0.09)	22
BIC-mCz	19.4	432/42	(0.16, 0.05)	33
mDBIC	13.5	431/42	(0.16, 0.05)	33
BOBO-Z	13.6	445/18	(0.15, 0.04)	24
BOBS-Z	26.9	456/23	(0.14, 0.06)	24
BSBS-Z	26.8	463/22	(0.13, 0.08)	24
ν-DABNA-O-Me	29.5	465/23	(0.13, 0.10)	51
TB-tCz	15.9	412/44	(0.17, 0.06)	53
TB-pCz	14.1	420/44	(0.17, 0.05)	53
t-DPAC-BN	21.6	460/28	(0.135, 0.094)	54
PAB	14.7	456/31	(0.145, 0.076)	55
2tPAB	16.8	456/27	(0.145, 0.076)	55
3tPAB	19.3	460/26	(0.141, 0.076)	55
B-O-dpa	16.3	443/43	(0.15, 0.05)	55
t-DAB-DPA	27.9	459/26	(0.13, 0.08)	69
CzBNO	13.6	454/36	(0.14, 0.08)	70
BN1	31.2	457/28	(0.14, 0.08)	23
BN3	37.6	458/23	(0.14, 0.08)	23
BFCz-DABNA	28.0	463/26	(0.13, 0.09)	71
4F-*ν*-DABNA	35.8	464/18	(0.13, 0.08)	25
4F-m-*ν*-DABNA	33.7	461/18	(0.13, 0.06)	25
1B-DTACrs	1.3	440/30	(0.15, 0.049)	72
2B-DTACrs	14.8	447/26	(0.15, 0.044)	72
CzBO	13.4	448/30	(0.15, 0.05)	73

Emitter	EQE_{max} (%)	λ_{EL}/FWHM (nm)	CIE $_{(x, y)}$	Ref.
DtBuAc-DBT: α-3BNMes	14.6	443/49	(0.15, 0.10)	74

Table S5. Key electroluminescent data of OLED devices based on different DBCz-Mes doping concentrations.

Doping concentration	λ_{EL} * (nm)	EQE_{max} † (%)	PE_{max} ‡ (lm/W)	FWHM§ (nm)	CIE (x,y) ‖
1%	452	33.9	19.9	17	(0.144, 0.058)
2%	452	28.6	18.9	18	(0.144, 0.059)
3%	452	19.3	13.8	18	(0.145, 0.060)

*EL emission maximum, measured at 10 mA/cm^2. †Maximum external quantum efficiency. ‡Maximum power efficiency. §Full width at half-maximum of the EL spectrum. ‖Commission Internationale de L´Eclairage coordinates.

Table S6. Key electroluminescent data of OLED devices without sensitizer.

Doping concentration	λ_{EL}* (nm)	V_{on}† (V)	EQE_{max}‡ (%)	PE_{max}§ (lm/W)	FWHM‖ (nm)	CIE¶ (x,y)
1%	451	4.0	16.2	6.5	16	(0.147, 0.038)
2%	451	4.0	13.1	5.2	16	(0.146, 0.040)

*EL emission maximum, measured at 10 mA/cm^2. †Turn-on voltage, measured at 1 cd/m^2. ‡Maximum external quantum efficiency. §Maximum power efficiency. ‖Full width at half-maximum of the EL spectrum. ¶Commission Internationale de L´Eclairage coordinates.

Table S7. Summary of photophysical data of doped film of DBCz-Mes in α,β-ADN .

Composition	λ_{em} (nm)	Φ_{PL} (%)	FWHM (nm)	τ_p (ns)	τ_d (μs)	k_F (10^7 s^{-1})	k_{IC} (10^7 s^{-1})
α,β-ADN:DBCz-Mes	454	97	18	3.3	N/A	29.6	0.9

Table S8. Key electroluminescent data of the fluorescent OLED devices.

Emitter	λ_{EL}* (nm)	V_{on}† (V)	EQE_{max}‡ (%)	PE_{max}§ (lm/W)	FWHM‖ (nm)	CIE¶ (x,y)	LT_{97}# (h)
DBCz-Mes	454	2.7	10.3	6.1	18	(0.142, 0.057)	177.9
BCz-BN	490	2.7	9.0	16.4	28	(0.095, 0.406)	9.2
t-DABNA	460	2.7	9.2	7.7	27	(0.133, 0.096)	45.0

*EL emission maximum, measured at 10 mA/cm^2. †Turn-on voltage, measured at 1 cd/m^2. ‡Maximum external quantum efficiency. §Maximum power efficiency. ‖Full width at half-maximum of the EL spectrum. ¶Commission Internationale de L´Eclairage coordinates. #The time to reach 97% of the initial luminance L_0.

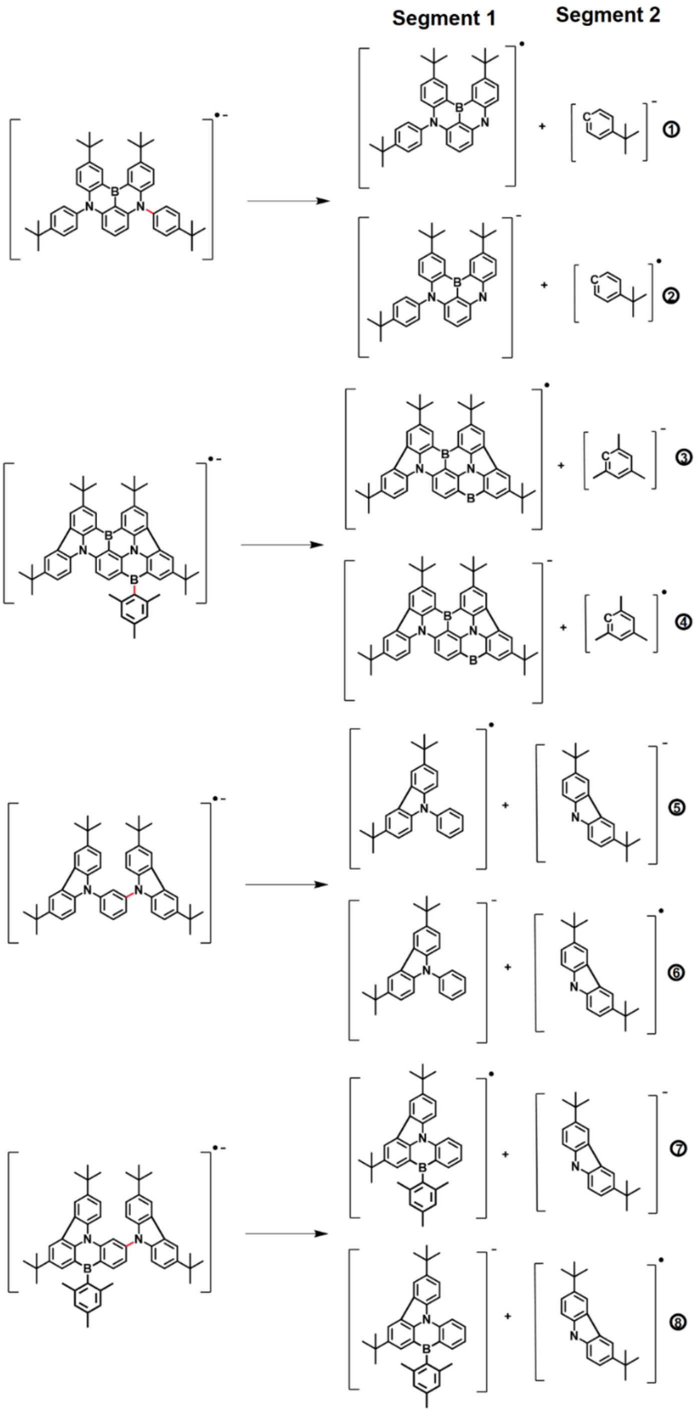

Table S9. BDE(-) values of C-X bonds (in red) in *t*-DABNA, DBCz-Mes, *t*-mCP and BIC-pCz with different dissociation pathways.

	1	*2*	3	4	5	6	7	8
**BDE(-) (eV)**	3.43	1.72	5.54	5.93	1.64	2.86	2.90	3.95

Table S10. Summary of reported fluorescent OLEDs based on deep blue emitters with CIE_y < 0.1.

Emitter	$EQE_{max/1k}$ (%)	$V_{on/1k}$ (V)	$PE_{max/1k}$ (%)	λ_{EL}/FWHM (nm)	CIE $_{(x, y)}$	L_0 (nit)	Lifetime (hr)	Ref.
**DBCz-Mes**	**10.3/9.4**	**2.7/4.6**	**6.1/3.7**	**454/18**	**(0.142, 0.057)**	**660**<br>**200**[*]	**LT_{97}: 178**<br>**LT_{97}: 1527**	**This work**
DABNA-1	5.4/4.9	-	-	-/25	(0.135, 0.072)	200	LT_{97}: 61	75
t-DABNA	7.6/7.0	-	-	-/24.7	(0.126, 0.098)	200	LT_{97}: 182	75
M-tDABNA	6.27/6.26	-	-	-/-	(0.137, 0.084)	400<br>200[*]	LT_{50}: 70<br>LT_{97}: 15	76
DABNA-NP-TB	7.03/7.0	2.6/3.6	-	458/23	(0.136, 0.076)	600<br>200[*]	LT_{95}: 85<br>LT_{97}: 368	77
SS	6.5/5.6	2.8/4.0	5.9/3.9	451/-	(0.15, 0.07)	-	-	61

[*]Converted using an acceleration factor n of 1.8.

Table S11. Crystal data and structure refinement for DBCz-Mes.

Empirical formula	$C_{55}H_{58}B_2N_2$
Formula weight	768.65
Temperature/K	179.99(10)
Crystal system	monoclinic
Space group	$P2_1/c$
a/Å	16.5951(6)
b/Å	13.6314(5)
c/Å	21.8830(8)
α/°	90
β/°	92.096(3)
γ/°	90
Volume/Å^3	4946.9(3)
Z	4
ρ_{calc}g/cm^3	1.032
μ/mm^{-1}	0.438
F(000)	1648.0
Crystal size/mm^3	0.25 × 0.2 × 0.15
Radiation	CuKα (λ = 1.54184)
2Θ range for data collection/°	7.642 to 153.292
Index ranges	$-20 \le h \le 20$, $-16 \le k \le 17$, $-27 \le l \le 27$
Reflections collected	39386
Independent reflections	9931 [R_{int} = 0.0489, R_{sigma} = 0.0490]
Data/restraints/parameters	9931/70/621
Goodness-of-fit on F^2	1.057
Final R indexes [I>=2σ (I)]	R_1 = 0.0799, wR_2 = 0.2427
Final R indexes [all data]	R_1 = 0.0971, wR_2 = 0.2614
Largest diff. peak/hole / e Å^{-3}	0.78/-0.22

REFERENCES AND NOTES

1. M. A. Baldo, D. F. O'Brien, Y. You, A. Shoustikov, S. Sibley, M. E. Thompson, S. R. Forrest, Highly efficient phosphorescent emission from organic electroluminescent devices. *Nature* **395**, 151–154 (1998).

2. Y. Ma, H. Zhang, J. Shen, C. Che, Electroluminescence from triplet metal–ligand charge-transfer excited state of transition metal complexes. *Synth. Met.* **94**, 245–248 (1998).

3. H. Uoyama, K. Goushi, K. Shizu, H. Nomura, C. Adachi, Highly efficient organic light-emitting diodes from delayed fluorescence. *Nature* **492**, 234–238 (2012).

4. S. Scholz, D. Kondakov, B. Lüssem, K. Leo, Degradation mechanisms and reactions in organic light-emitting devices. *Chem. Rev.* **115**, 8449–8503 (2015).

5. X. Cai, S.-J. Su, Marching toward highly efficient, pure-blue, and stable thermally activated delayed fluorescent organic light-emitting diodes. *Adv. Funct. Mater.* **28**, 1802558 (2018).

6. P.-T. Chou, Y. Chi, Phosphorescent dyes for organic light-emitting diodes. *Chemistry* **13**, 380–395 (2007).

7. H. Yokoyama, Physics and device applications of optical microcavities. *Science* **256**, 66–70 (1992).

8. D. Poitras, C.-C. Kuo, C. Py, Design of high-contrast OLEDs with microcavity effect. *Opt. Express* **16**, 8003–8015 (2008).

9. S. Madayanad Suresh, D. Hall, D. Beljonne, Y. Olivier, E. Zysman-Colman, Multiresonant thermally activated delayed fluorescence emitters based on heteroatom-doped nanographenes: Recent advances and prospects for organic light-emitting diodes. *Adv. Funct. Mater.* **30**, 1908677 (2020).

10. J. M. Ha, S. H. Hur, A. Pathak, J.-E. Jeong, H. Y. Woo, Recent advances in organic luminescent materials with narrowband emission. *NPG Asia Mater.* **13**, 53 (2021).

11. S. S. Kothavale, J. Y. Lee, Three- and four-coordinate, boron-based, thermally activated delayed fluorescent emitters. *Adv. Opt. Mater.* **8**, 2000922 (2020).

12. H. J. Kim, T. Yasuda, Narrowband emissive thermally activated delayed fluorescence materials. *Adv. Opt. Mater.* **10**, 2201714 (2022).

13. A. Pershin, D. Hall, V. Lemaur, J.-C. Sancho-Garcia, L. Muccioli, E. Zysman-Colman, D. Beljonne, Y. Olivier, Highly emissive excitons with reduced exchange energy in thermally activated delayed fluorescent molecules. *Nat. Commun.* **10**, 597 (2019).

14. K. Matsui, S. Oda, K. Yoshiura, K. Nakajima, N. Yasuda, T. Hatakeyama, One-shot multiple borylation toward BN-doped nanographenes. *J. Am. Chem. Soc.* **140**, 1195–1198 (2018).

15. S. M. Suresh, E. Duda, D. Hall, Z. Yao, S. Bagnich, A. M. Z. Slawin, H. Bässler, D. Beljonne, M. Buck, Y. Olivier, A. Köhler, E. Zysman-Colman, A deep blue B,N-doped heptacene emitter that shows both thermally activated selayed fluorescence and delayed fluorescence by triplet–triplet annihilation. *J. Am. Chem. Soc.* **142**, 6588–6599 (2020).

16. S. Oda, B. Kawakami, R. Kawasumi, R. Okita, T. Hatakeyama, Multiple resonance effect-induced sky-blue thermally activated delayed fluorescence with a narrow emission band. *Org. Lett.* **21**, 9311–9314 (2019).

17. Y. Kondo, K. Yoshiura, S. Kitera, H. Nishi, S. Oda, H. Gotoh, Y. Sasada, M. Yanai, T. Hatakeyama, Narrowband deep-blue organic light-emitting diode featuring an organoboron-based emitter. *Nat. Photonics* **13**, 678–682 (2019).

18. Y. Zhang, G. Li, L. Wang, T. Huang, J. Wei, G. Meng, X. Wang, X. Zeng, D. Zhang, L. Duan, Fusion of multi-resonance fragment with conventional polycyclic aromatic hydrocarbon for nearly BT.2020 green emission. *Angew. Chem. Int. Ed. Engl.* **61**, e202202380 (2022).

19. J. Gierschner, H.-G. Mack, L. Lüer, D. Oelkrug, Fluorescence and absorption spectra of oligophenylenevinylenes: Vibronic coupling, band shapes, and solvatochromism. *J. Chem. Phys.* **116**, 8596–8609 (2002).

20. G. Meng, D. Zhang, J. Wei, Y. Zhang, T. Huang, Z. Liu, C. Yin, X. Hong, X. Wang, X. Zeng, D. Yang, D. Ma, G. Li, L. Duan, Highly efficient and stable deep-blue OLEDs based on narrowband emitters featuring an orthogonal spiro-configured indolo[3,2,1-*de*]acridine structure. *Chem. Sci.* **13**, 5622–5630 (2022).

21. J. J. Hunter, J. I. W. Morgan, W. H. Merigan, D. H. Sliney, J. R. Sparrow, D. R. Williams, The susceptibility of the retina to photochemical damage from visible light. *Prog. Retin. Eye Res.* **31**, 28–42 (2012).

22. T. Hatakeyama, K. Shiren, K. Nakajima, S. Nomura, S. Nakatsuka, K. Kinoshita, J. Ni, Y. Ono, T. Ikuta, Ultrapure blue thermally activated delayed fluorescence molecules: Efficient HOMO–LUMO separation by the multiple resonance effect. *Adv. Mater.* **28**, 2777–2781 (2016).

23. X. Lv, J. Miao, M. Liu, Q. Peng, C. Zhong, Y. Hu, X. Cao, H. Wu, Y. Yang, C. Zhou, J. Ma, Y. Zou, C. Yang, Extending the π-skeleton of multi-resonance TADF materials towards high-efficiency narrowband deep-blue emission. *Angew. Chem. Int. Ed. Engl.* **61**, e202201588 (2022).

24. I. S. Park, M. Yang, H. Shibata, N. Amanokura, T. Yasuda, Achieving ultimate narrowband and ultrapure blue organic light-emitting diodes based on polycyclo-heteraborin multi-resonance delayed-fluorescence emitters. *Adv. Mater.* **34**, e2107951 (2022).

25. K. Rayappa Naveen, H. Lee, R. Braveenth, K. Joon Yang, S. Jae Hwang, J. Hyuk Kwon, Deep blue diboron embedded multi-resonance thermally activated delayed fluorescence emitters for narrowband organic light emitting diodes. *Chem. Eng. J.* **432**, 134381 (2022).

26. S. Oda, B. Kawakami, Y. Yamasaki, R. Matsumoto, M. Yoshioka, D. Fukushima, S. Nakatsuka, T. Hatakeyama, One-shot synthesis of expanded heterohelicene exhibiting narrowband thermally activated delayed fluorescence. *J. Am. Chem. Soc.* **144**, 106–112 (2022).

27. C. Cao, J.-H. Tan, Z.-L. Zhu, J.-D. Lin, H.-J. Tan, H. Chen, Y. Yuan, M.-K. Tse, W.-C. Chen, C.-S. Lee, Intramolecular cyclization: A convenient strategy to realize efficient BT.2020 blue multi-resonance emitter for organic light-emitting diodes. *Angew. Chem. Int. Ed.* **62**, e202215226 (2023).

28. X. Li, Y.-Z. Shi, K. Wang, M. Zhang, C.-J. Zheng, D.-M. Sun, G.-L. Dai, X.-C. Fan, D.-Q. Wang, W. Liu, Y.-Q. Li, J. Yu, X.-M. Ou, C. Adachi, X.-H. Zhang, Thermally activated delayed fluorescence carbonyl derivatives for organic light-emitting diodes with extremely narrow full width at half-maximum. *ACS Appl. Mater. Interfaces* **11**, 13472–13480 (2019).

29. M. Hirai, N. Tanaka, M. Sakai, S. Yamaguchi, Structurally constrained boron-, nitrogen-, silicon-, and phosphorus-centered polycyclic π-conjugated systems. *Chem. Rev.* **119**, 8291–8331 (2019).

30. Z. Zhou, A. Wakamiya, T. Kushida, S. Yamaguchi, Planarized triarylboranes: Stabilization by structural constraint and their plane-to-bowl conversion. *J. Am. Chem. Soc.* **134**, 4529–4532 (2012).

31. S. Saito, K. Matsuo, S. Yamaguchi, Polycyclic π-electron system with boron at its center. *J. Am. Chem. Soc.* **134**, 9130–9133 (2012).

32. M. Ando, M. Sakai, N. Ando, M. Hirai, S. Yamaguchi, Planarized *B,N*-phenylated dibenzoazaborine with a carbazole substructure: Electronic impact of the structural constraint. *Org. Biomol. Chem.* **17**, 5500–5504 (2019).

33. X. Wang, Y. Zhang, H. Dai, G. Li, M. Liu, G. Meng, X. Zeng, T. Huang, L. Wang, Q. Peng, D. Yang, D. Ma, D. Zhang, L. Duan, Mesityl-functionalized multi-resonance organoboron delayed fluorescent frameworks with wide-range color tunability for narrowband OLEDs. *Angew. Chem. Int. Ed.* **61**, e202206916 (2022).

34. G. Liu, H. Sasabe, K. Kumada, A. Matsunaga, H. Katagiri, J. Kido, Facile synthesis of multi-resonance ultra-pure-green TADF emitters based on bridged diarylamine derivatives for efficient OLEDs with narrow emission. *J. Mater. Chem. C* **9**, 8308–8313 (2021).

35. X.-C. Fan, K. Wang, Y.-Z. Shi, Y.-C. Cheng, Y.-T. Lee, J. Yu, X.-K. Chen, C. Adachi, X.-H. Zhang, Ultrapure green organic light-emitting diodes based on highly distorted fused π-conjugated molecular design. *Nat. Photonics* **17**, 280–285 (2023).

36. M. Yang, I. S. Park, T. Yasuda, Full-color, narrowband, and high-efficiency electroluminescence from boron and carbazole embedded polycyclic heteroaromatics. *J. Am. Chem. Soc.* **142**, 19468–19472 (2020).

37. Z. Shuai, Thermal vibration correlation function formalism for molecular excited state decay rates. *Chinese J. Chem.* **38**, 1223–1232 (2020).

38. Z. Shuai, Q. Peng, Excited states structure and processes: Understanding organic light-emitting diodes at the molecular level. *Phys. Rep.* **537**, 123–156 (2014).

39. Y. Yang, Y. Zheng, W. Cao, A. Titov, J. Hyvonen, J. R. Manders, J. Xue, P. H. Holloway, L. Qian, High-efficiency light-emitting devices based on quantum dots with tailored nanostructures. *Nat. Photonics* **9**, 259–266 (2015).

40. K. Zhang, D. Peng, K. M. Lau, Z. Liu, Fully-integrated active matrix programmable UV and blue micro-LED display system-on-panel (SoP). *J. Soc. Inf. Disp.* **25**, 240–248 (2017).

41. H. He, S. Mei, Z. Wen, D. Yang, B. Yang, W. Zhang, F. Xie, G. Xing, R. Guo, Recent advances in blue perovskite quantum dots for light-emitting diodes. *Small* **18**, 2103527 (2022).

42. J. Liu, Y. Zhu, T. Tsuboi, C. Deng, W. Lou, D. Wang, T. Liu, Q. Zhang, Toward a BT.2020 green emitter through a combined multiple resonance effect and multi-lock strategy. *Nat. Commun.* **13**, 4876 (2022).

43. M. Yang, S. Shikita, H. Min, I. S. Park, H. Shibata, N. Amanokura, T. Yasuda, Wide-range color tuning of narrowband emission in multi-resonance organoboron delayed fluorescence materials through rational imine/amine functionalization. *Angew. Chem. Int. Ed. Engl.* **60**, 23142–23147 (2021).

44. T. Huang, Q. Wang, S. Xiao, D. Zhang, Y. Zhang, C. Yin, D. Yang, D. Ma, Z. Wang, L. Duan, Simultaneously enhanced reverse intersystem crossing and radiative decay in thermally activated delayed fluorophors with multiple through-space charge transfers. *Angew. Chem. Int. Ed. Engl.* **60**, 23771–23776 (2021).

45. C. Yin, D. Zhang, Y. Zhang, Y. Lu, R. Wang, G. Li, L. Duan, High-efficiency narrow-band electro-fluorescent devices with thermally activated delayed fluorescence sensitizers combined through-bond and through-space charge transfers. *CCS Chem.* **2**, 1268–1277 (2020).

46. C.-Y. Chan, M. Tanaka, Y.-T. Lee, Y.-W. Wong, H. Nakanotani, T. Hatakeyama, C. Adachi, Stable pure-blue hyperfluorescence organic light-emitting diodes with high-efficiency and narrow emission. *Nat. Photonics* **15**, 203–207 (2021).

47. S. O. Jeon, K. H. Lee, J. S. Kim, S.-G. Ihn, Y. S. Chung, J. W. Kim, H. Lee, S. Kim, H. Choi, J. Y. Lee, High-efficiency, long-lifetime deep-blue organic light-emitting diodes. *Nat. Photonics* **15**, 208–215 (2021).

48. R. Braveenth, H. Lee, J. D. Park, K. J. Yang, S. J. Hwang, K. R. Naveen, R. Lampande, J. H. Kwon, Achieving narrow FWHM and high EQE over 38% in blue OLEDs using rigid heteroatom-based deep blue TADF sensitized host. *Adv. Funct. Mater.* **31**, 2105805 (2021).

49. K. R. Naveen, H. Lee, R. Braveenth, D. Karthik, K. J. Yang, S. J. Hwang, J. H. Kwon, Achieving high efficiency and pure blue color in hyperfluorescence organic light emitting diodes using organo-boron based emitters. *Adv. Funct. Mater.* **32**, 2110356 (2022).

50. D. Zhang, X. Song, A. J. Gillett, B. H. Drummond, S. T. E. Jones, G. Li, H. He, M. Cai, D. Credgington, L. Duan, Efficient and stable deep-blue fluorescent organic light-emitting diodes employing a sensitizer with fast triplet upconversion. *Adv. Mater.* **32**, 1908355 (2020).

51. H. Tanaka, S. Oda, G. Ricci, H. Gotoh, K. Tabata, R. Kawasumi, D. Beljonne, Y. Olivier, T. Hatakeyama, Hypsochromic shift of multiple-resonance-induced thermally activated delayed

fluorescence by oxygen atom incorporation. *Angew. Chem. Int. Ed. Engl.* **60**, 17910–17914 (2021).

52. Y. Qiu, H. Xia, J. Miao, Z. Huang, N. Li, X. Cao, J. Han, C. Zhou, C. Zhong, C. Yang, Narrowing the electroluminescence spectra of multiresonance emitters for high-performance blue OLEDs by a peripheral decoration strategy. *ACS Appl. Mater. Interfaces* **13**, 59035–59042 (2021).

53. H. J. Kim, H. Kang, J.-E. Jeong, S. H. Park, C. W. Koh, C. W. Kim, H. Y. Woo, M. J. Cho, S. Park, D. H. Choi, Ultra-deep-blue aggregation-induced delayed fluorescence emitters: Achieving nearly 16% EQE in solution-processed nondoped and doped OLEDs with $CIE_y < 0.1$. *Adv. Funct. Mater.* **31**, 2102588 (2021).

54. Y. Wang, K. Di, Y. Duan, R. Guo, L. Lian, W. Zhang, L. Wang, The selective regulation of borylation site based on one-shot electrophilic C–H borylation reaction, achieving highly efficient narrowband organic light-emitting diodes. *Chem. Eng. J.* **431**, 133221 (2022).

55. Y. Wang, Y. Duan, R. Guo, S. Ye, K. Di, W. Zhang, S. Zhuang, L. Wang, A periphery cladding strategy to improve the performance of narrowband emitters, achieving deep-blue OLEDs with $CIE_y < 0.08$ and external quantum efficiency approaching 20%. *Org. Electron.* **97**, 106275 (2021).

56. J. Park, J. Lim, J. H. Lee, B. Jang, J. H. Han, S. S. Yoon, J. Y. Lee, Asymmetric blue multiresonance TADF emitters with a narrow emission band. *ACS Appl. Mater. Interfaces* **13**, 45798–45805 (2021).

57. L. Su, F. Cao, C. Cheng, T. Tsuboi, Y. Zhu, C. Deng, X. Zheng, D. Wang, Z. Liu, Q. Zhang, High fluorescence rate of thermally activated delayed fluorescence emitters for efficient and stable blue OLEDs. *ACS Appl. Mater. Interfaces* **12**, 31706–31715 (2020).

58. M. Hong, M. K. Ravva, P. Winget, J.-L. Brédas, Effect of substituents on the electronic structure and degradation process in carbazole derivatives for blue OLED host materials. *Chem. Mater.* **28**, 5791–5798 (2016).

59. R. Wang, Y.-L. Wang, N. Lin, R. Zhang, L. Duan, J. Qiao, Effects of ortho-linkages on the molecular stability of organic light-emitting diode materials. *Chem. Mater.* **30**, 8771–8781 (2018).

60. J. Sun, H. Ahn, S. Kang, S.-B. Ko, D. Song, H. A. Um, S. Kim, Y. Lee, P. Jeon, S.-H. Hwang, Y. You, C. Chu, S. Kim, Exceptionally stable blue phosphorescent organic light-emitting diodes. *Nat. Photonics* **16**, 212–218 (2022).

61. R. Wang, Q.-Y. Meng, Y.-L. Wang, J. Qiao, Negative charge management to make fragile bonds less fragile toward electrons for robust organic optoelectronic materials. *CCS Chem.* **4**, 331–343 (2022).

62. P. Sun, D. Liu, F. Zhu, D. Yan, An efficient solid-solution crystalline organic light-emitting diode with deep-blue emission. *Nat. Photonics* **17**, 264–272 (2023).

63. O. V. Dolomanov, L. J. Bourhis, R. J. Gildea, J. A. K. Howard, H. Puschmann, *OLEX2*: A complete structure solution, refinement and analysis program. *J. Appl. Cryst.* **42**, 339–341 (2009).

64. G. M. Sheldrick, SHELXT—Integrated space-group and crystal-structure determination. *Acta Crystallogr. A Found. Adv.* **71**, 3–8 (2015).

65. G. M. Sheldrick, Crystal structure refinement with SHELXL. *Acta Crystallogr. C Struct. Chem.* **71**, 3–8 (2015).

66. M. J. Frisch, G. W. Trucks, H. B. Schlegel, G. E. Scuseria, M. A. Robb, J. R. Cheeseman, G. Scalmani, V. Barone, G. A. Petersson, H. Nakatsuji, X. Li, M. Caricato, A. V. Marenich, J. Bloino, B. G. Janesko, R. Gomperts, B. Mennucci, H. P. Hratchian, J. V. Ortiz, A. F. Izmaylov, J. L. Sonnenberg, Williams, F. Ding, F. Lipparini, F. Egidi, J. Goings, B. Peng, A. Petrone, T. Henderson, D. Ranasinghe, V. G. Zakrzewski, J. Gao, N. Rega, G. Zheng, W. Liang, M. Hada, M. Ehara, K. Toyota, R. Fukuda, J. Hasegawa, M. Ishida, T. Nakajima, Y. Honda, O. Kitao, H. Nakai, T. Vreven, K. Throssell, J. A. Montgomery Jr., J. E. Peralta, F. Ogliaro, M. J. Bearpark, J. J. Heyd, E. N. Brothers, K. N. Kudin, V. N. Staroverov, T. A.

Keith, R. Kobayashi, J. Normand, K. Raghavachari, A. P. Rendell, J. C. Burant, S. S. Iyengar, J. Tomasi, M. Cossi, J. M. Millam, M. Klene, C. Adamo, R. Cammi, J. W. Ochterski, R. L. Martin, K. Morokuma, O. Farkas, J. B. Foresman, D. J. Fox., Gaussian 16 (Wallingford, 2016).

67. C. Adamo, V. Barone, Toward reliable density functional methods without adjustable parameters: The PBE0 model. *J. Chem. Phys.* **110**, 6158–6170 (1999).

68. W. Humphrey, A. Dalke, K. Schulten, VMD: Visual molecular dynamics. *J. Mol. Graph.* **14**, 33–38 (1996).

69. J. H. Kim, W. J. Chung, J. Kim, J. Y. Lee, Concentration quenching–resistant multiresonance thermally activated delayed fluorescence emitters. *Mater. Today Energy* **21**, 100792 (2021).

70. J. Han, Z. Huang, X. Lv, J. Miao, Y. Qiu, X. Cao, C. Yang, Simple molecular design strategy for multiresonance induced TADF emitter: Highly efficient deep blue to blue electroluminescence with high color purity. *Adv. Opt. Mater.* **10**, 2102092 (2022).

71. H. Lee, R. Braveenth, J. D. Park, C. Y. Jeon, H. S. Lee, J. H. Kwon, Manipulating spectral width and emission wavelength towards highly efficient blue asymmetric carbazole fused multi-resonance emitters. *ACS Appl. Mater. Interfaces* **14**, 36927–36935 (2022).

72. C.-Y. Chan, S. Madayanad Suresh, Y.-T. Lee, Y. Tsuchiya, T. Matulaitis, D. Hall, A. M. Z. Slawin, S. Warriner, D. Beljonne, Y. Olivier, C. Adachi, E. Zysman-Colman, Two boron atoms *versus* one: High-performance deep-blue multi-resonance thermally activated delayed fluorescence emitters. *Chem. Commun.* **58**, 9377–9380 (2022).

73. I. S. Park, H. Min, T. Yasuda, Ultrafast Triplet–Singlet Exciton Interconversion in Narrowband Blue Organoboron Emitters Doped with Heavy Chalcogens. *Angew. Chem. Int. Ed. Engl.* **61**, e202205684 (2022).

74. K. Stavrou, S. Madayanad Suresh, D. Hall, A. Danos, N. A. Kukhta, A. M. Z. Slawin, S. Warriner, D. Beljonne, Y. Olivier, A. Monkman, E. Zysman-Colman, Emission and

absorption tuning in TADF B,N-doped heptacenes: Toward ideal-blue hyperfluorescent OLEDs. *Adv. Opt. Mater.* **10**, 2200688 (2022).

75. K. H. Lee, J. Y. Lee, Paradigm change of blue emitters: Thermally activated fluorescence emitters as long-living fluorescence emitters by triplet exciton quenching. *Org. Electron.* **75**, 105377 (2019).

76. H. Lim, S.-J. Woo, Y. H. Ha, Y.-H. Kim, J.-J. Kim, Breaking the efficiency limit of deep-blue fluorescent OLEDs based on anthracene derivatives. *Adv. Mater.* **34**, 2100161 (2022).

77. S. M. Cho, K. M. Youn, H. I. Yang, S. H. Lee, K. R. Naveen, D. Karthik, H. Jeong, J. H. Kwon, Anthracene-dibenzofuran based electron transport type hosts for long lifetime multiple resonance pure blue OLEDs. *Org. Electron.* **105**, 106501 (2022).
